# Paired single-cell and spatial transcriptional profiling reveals a central osteopontin macrophage response mediating tuberculous granuloma formation

**DOI:** 10.1128/mbio.01559-25

**Published:** 2025-08-07

**Authors:** Charlie J. Pyle, Liuyang Wang, Rebecca W. Beerman, Vaibhav Jain, Henry K. E. Ohman, Brandon A. Thompson, Karen R. Abramson, Dennis C. Ko, Simon G. Gregory, Clare M. Smith, Jadee L. Neff, Rebecca J. Richardson, Jason E. Stout, David M. Tobin

**Affiliations:** 1 Department of Molecular Genetics and Microbiology, Duke University School of Medicine 12277 , Durham, North Carolina, USA; 2 Department of Integrative Immunobiology, Duke University School of Medicine 12277 , Durham, North Carolina, USA; 3 Duke Molecular Physiology Institute, Duke University 3065, Durham, North Carolina, USA; 4 Department of Pathology, Duke University School of Medicine 12277 , Durham, North Carolina, USA; 5 School of Physiology, Pharmacology and Neuroscience, University of Bristol 1980, Bristol, United Kingdom; 6 Department of Medicine, Division of Infectious Diseases, Duke University School of Medicine 12277 , Durham, North Carolina, USA; The Hebrew University of Jerusalem, Rehovot, Israel

**Keywords:** tuberculosis, granuloma, *Mycobacterium tuberculosis*, macrophage, spatial transcriptomics, osteopontin, spp1, scRNA-seq

## Abstract

Granulomas are classic manifestations of tuberculosis pathogenesis. They result from an ensemble of immune responses to *Mycobacterium tuberculosis* infection, but the identities, arrangement, cellular interactions, and regulation of the cells that comprise them have thus far been incompletely understood. To better understand the composition of granulomas, we conducted spatial and single-cell RNA sequencing of granulomas in biopsy specimens from patients with tuberculosis. We found that granulomas consist of concentric transcriptional laminae surrounding areas of central necrosis. We identified distinct populations of granuloma-associated stromal cells, fibroblasts, lymphocytes, mast cells, dendritic cells, neutrophils, and macrophages. Furthermore, gene expression among these cell populations differed by location within granulomas. We used inferential analysis to predict dominant granuloma cell-cell interactions, the activity of major signaling pathways, and transcription factor activities. Using spatial deconvolution, we mapped a conserved pattern of cellular organization dominated by macrophages rich in *SPP1*/osteopontin expression. Trajectory analysis of macrophage subtypes mapped their differentiation and supported the importance of SPP1 to granuloma macrophage polarization. Using the *Mycobacterium marinum*-zebrafish model, we found that mycobacterial infection induces *spp1* expression in macrophages and that *spp1* ablation results in granuloma formation defects and reduced survival in adult animals. Cumulatively, we have identified a dominant macrophage granuloma population as well as its central regulatory gene in human samples and confirmed the importance of *spp1* to granuloma biology *in vivo*.

**IMPORTANCE:**

Tuberculosis is the world’s most deadly single-pathogen infection. Its causative bacterium, *Mycobacterium tuberculosis*, sickens over 10 million people annually. Mycobacterial granulomas are the pathological hallmark of the infection and are critical determinants of disease trajectory. Granulomas form as a physiological barrier to contain infected macrophages and reduce bacterial dissemination. However, that barrier also reduces access of antibiotics and mycobactericidal immune cells to the pathogen, thereby promoting chronic infection and end-organ damage. This work supplies the field with a map of the conserved features of human tuberculosis granulomas and provides a valuable resource for future exploration of critical factors in tuberculosis pathogenesis, exemplified here by functional findings around the roles of *spp1*/osteopontin-expressing macrophages in mycobacterial granulomas.

## INTRODUCTION

Tuberculosis (TB) is a pulmonary infection caused by inhalation of aerosolized respiratory droplets that contain the obligate human pathogen *Mycobacterium tuberculosis* (*M. tb*). After exposure, approximately 10% of infected individuals develop a protracted infection characterized by the formation of chronic granulomatous pulmonary lesions (1). Alveolar macrophages that encounter *M. tb* in the lung are not able to eradicate it and become the bacteria’s initial intracellular niche after phagocytosis (2). Granulomas originate from nests of infected macrophages and progress into heterogeneous organizations of lesion-adapted immune and stromal cells that fully mature with the onset of adaptive immunity. These complex cellular communities can function to segregate *M. tb* away from vulnerable host tissues, thereby slowing the dissemination of the infection, or depending on context, serve as a dispersal point for infected macrophages (3, 4). Unfortunately, this restrictive *de novo* tissue compartment also protects the bacteria from mycobactericidal immune cells and antimicrobials (5, 6).

Granuloma biology has been an ongoing area of intense research for decades, incorporating observations from human tissues (7–10) as well as numerous animal models (11–16). Recent studies have characterized the cell populations of mycobacterial granulomas in macaques (17) and zebrafish (18) as well as the spatial distribution of gene transcripts or protein within rabbit, macaque, and human granulomas (19–21). However, the location-specific gene expression patterns and unique cellular constituencies that sustain the pathology of *M. tb* granulomas in human lungs have remained enigmatic. Here we provide a transcriptional blueprint of the human TB granuloma as determined by paired spatial and single-cell sequencing from a clinical biopsy library of *M. tb-*infected tissue. We determined the cell populations that make up granulomas and established their arrangement within specific lesions. We also predicted the regulatory and functional consequences of cell-specific and structurally emergent expression patterns in the integrated data.

Heterogeneous macrophage polarization states and cellular responses to mycobacteria are central to TB granuloma biology (22–24). In human alveolar macrophages and granulomas, *M. tb* infection induces the expression of *SPP1*, encoding osteopontin (OPN) (25). OPN is a marker of disease severity in TB (26) and a conserved component of granulomatous diseases (27). From paired transcriptional profiling, we identified specific granuloma macrophage subsets that differentially express *SPP1* and discovered that they represent the most prevalent granuloma macrophage population in these samples. Then, using zebrafish *Mycobacterium marinum* (*M. marinum*) granuloma models, we identified an essential role for *spp1* in lesion formation and function. These results and data sets provide a detailed view of the cellular organization and molecular foundations of human tuberculous granulomas.

## RESULTS

### Granuloma specimens

**Fig 1:**
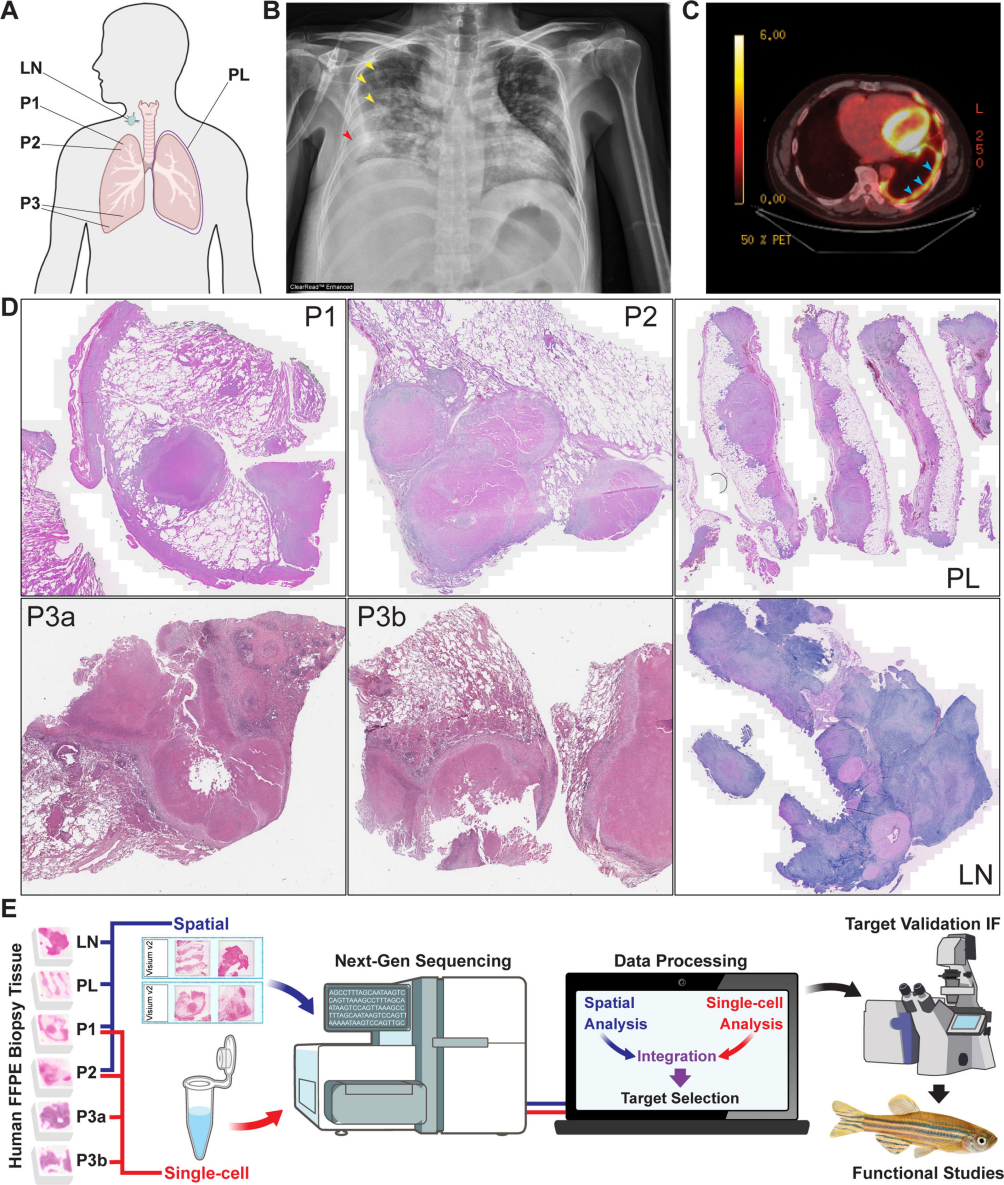
Tissue specimens containing *M. tb* granulomas were collected from five different TB patients. (**A**) Locations from which tissue resection specimens were obtained. (**B**) A chest radiograph of patient P1 revealed numerous pulmonary nodules (yellow arrows) and a loculated right pleural effusion (red arrow). (**C**) A PET-CT scan of patient PL demonstrated a small left pleural effusion with multiple pleural nodules (blue arrows) demonstrating increased uptake of 18-fluoroxyglucose. (**D**) H&E-stained sections from tissue biopsy sections included in spatial and single-cell sequencing experiments. (**E**) Schematic of the approach to transcriptional analysis of TB granuloma biopsies and subsequent validation studies.

We identified formalin-fixed, paraffin-embedded (FFPE) clinical biopsy specimens resected from individuals diagnosed with TB. We selected biopsies from five different culture-proven TB cases representing diverse disease presentations, including necrotic pulmonary (P1, P2, or P3), pleural (PL), or right supraclavicular lymph node (LN) disease (Fig. 1A). Typical findings of pulmonary consolidation on plain radiographs (Fig. 1B) and hypermetabolic pleural nodules (Fig. 1C) are illustrated, respectively. All tissue specimens were diagnostic biopsies and obtained prior to any antimycobacterial treatment. Histopathology of the resected tissues demonstrated necrotizing granulomatous inflammation. Mycobacterial genotype was determined by spoligotyping/MIRU. To maximize the translational applicability of our findings, we selected granulomas from infections with three globally dispersed, clinically relevant *M. tb* strains (28). Cultures were susceptible to all first-line antituberculosis drugs except for *M. tb* from P1, which was resistant to pyrazinamide (Table 1). Evaluation of samples P1, P2, PL, LN, P3a, and P3b uncovered 34 granulomas with a gross pathology of concentric tissue layers surrounding a central focus (Fig. 1D).

**TABLE 1:** Clinical data associated with the granuloma specimens

Parameter	P1	P2	P3	PL	LN
Sex	Male	Male	Male	Male	Male
Age	39 years	69 years	53 years	52 years	37 years
Country of origin	India	South Korea	USA	Mexico	India
Race/ethnicity	Asian	Asian	White	Hispanic	Asian
Comorbidities	None	Hypertension	Hypertension, type 2 diabetes, sleep apnea, nephrolithiasis	Rheumatic mitral stenosis status post-mitral valve replacement, atrial fibrillation, type 2 diabetes, gout, hypertension	None
Tissue	Lung	Lung	Lung	Pleura	Lymph node
Location	Right upper lobe	Right upper lobe	Right lower lobe	Left pleura	Right supraclavicular
*M. tb* lineage	L1	L2	L4	L4	L1
First-line resistance	Pyrazinamide	None	None	None	None

Our observations are consistent with historical analysis of human TB granulomas (7), but uncovered sample-specific pathology across the 34 granulomas, indicative of different stages of infection. Sample P1 showed intermediate-stage granuloma formation with multiple large, coalescing granulomas with well-developed central caseating necrosis. A few neutrophils were seen at the boundary between necrotic and viable tissue, followed by a thin rim of fibroblasts, histiocytes, lymphocytes, and vascular proliferation in a pattern resembling granulation tissue. Sample P2 showed late-stage granuloma formation. There were also multiple large coalescing granulomas in P2, but in that sample, there was early collagen deposition within the necrotic center and the thickened fibrotic rim. A few multinucleated giant cells were noted, but only scattered lymphocytes and occasional vessels were observed in the fibrotic rim. Sample PL showed very early granuloma formation, characterized by multiple small fibrohistiocytic nodules containing scattered lymphocytes and occasional giant cells. Only two of the granulomas showed a very small focus of central necrosis. LN showed several intermediate stage granulomas similar to those described in P1, as well as a few very old granulomas characterized by hyalinized nodules with scant fibroblast cellularity and occasional central necrosis. Histologic examination of P3a and P3b showed three extremely large granulomas ranging in size from 1.2 cm to 1.3 cm in greatest dimension. The large granulomas were late stage, with histologic features like those seen in sample P2. Three smaller granulomas in the samples were early stage, histologically similar to those described in PL. Using these TB granuloma biopsy specimens, we devised a systematic approach to uncover and evaluate fundamental constituents of mycobacterial granuloma pathogenesis (Fig. 1E).

### Spatial transcriptomics of human *M. tb* granulomas

**Fig 2:**
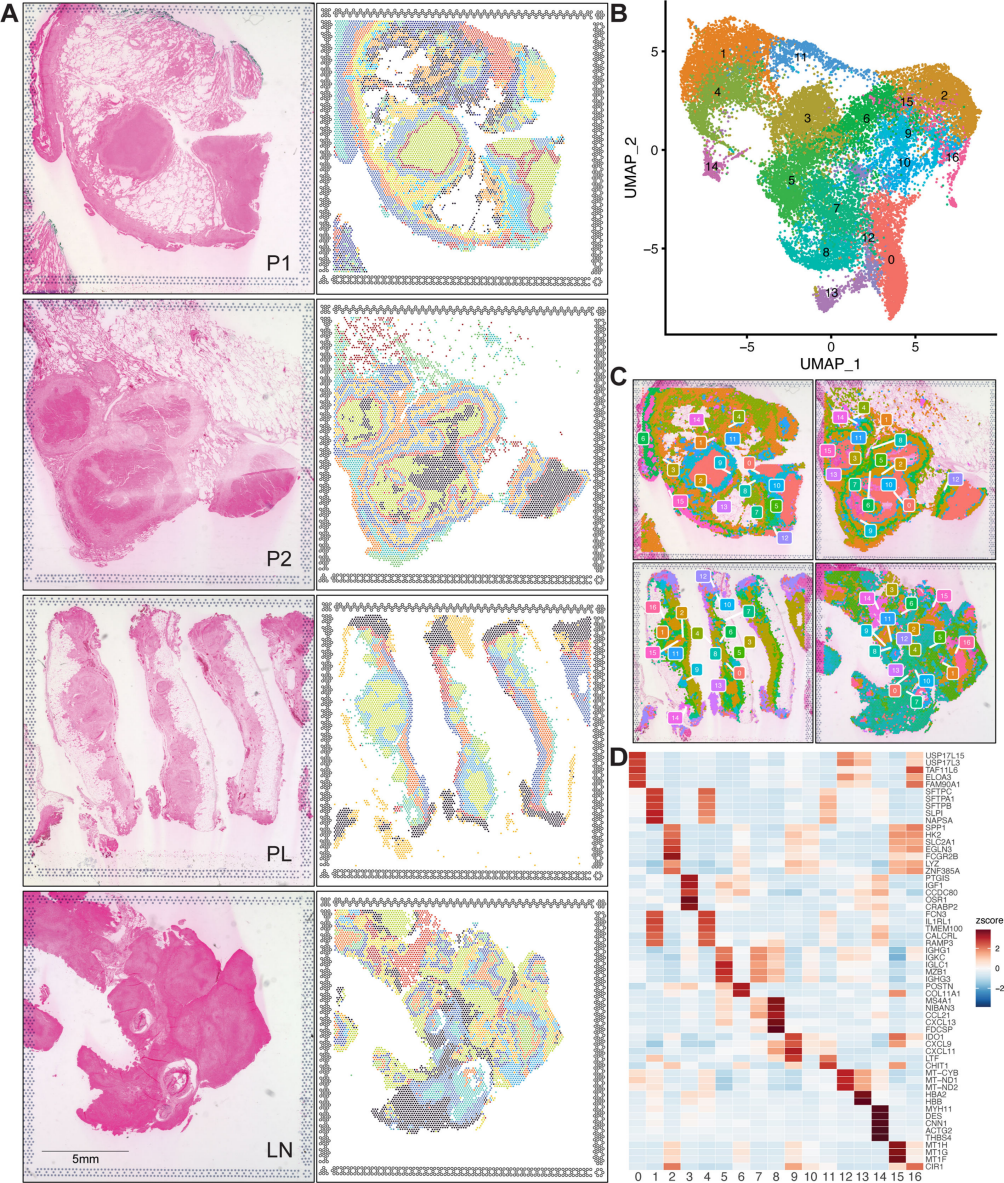
The spatial transcriptomes of four granuloma tissue specimens (P1, P2, PL and LN) were assayed with 10× Genomics Visium v2. (**A**) Eosin staining and unsupervised clustering of 55 µM spots in individual tissue sections. (**B**) UMAP plot of spatial clusters generated by unsupervised clustering of combined spatial data (**C**) reveals 17 global transcriptional regions between granulomas. (**D**) Heatmap plot shows the average expression of the top five differentially expressed genes for each cluster. The average gene expression values were further z-score normalized across clusters.

To examine the interdependence of lesional architecture and gene expression, we first evaluated the whole spatial transcriptomes of samples P1, P2, PL, and LN at 55 µM resolution (~5–10 cells) using the 10× Genomics Visium v2 with CytAssist assay. Unsupervised clustering and analysis of positionally segregated mRNA levels revealed serial transcriptional laminae occupying distinct anatomical regions of tissue granulomas (Fig. 2A; Fig. S1). Regional gene signatures varied between samples, but a consistent pattern of paired transcriptional and histologic features emerged in necrotic granulomas (P1, P2, and LN). PL granulomas had cellular cores and thus provided a non-necrotic set of lesions for comparison. Despite variability in clusters between samples, we identified 17 conserved regions of differential expression in our samples (Fig. 2B and C; Fig. S2) with unique transcriptional signatures (Fig. 2D; Fig. S2E, Table S1) and putative functional response cassettes (Fig. S3).

These clusters represent transcriptional neighborhoods and were associated with readily identifiable histological hallmarks and characteristic expression profiles, including vasculature (Cluster 14), blood (Cluster 13), necrotic tissue (Clusters 0 and 12), and lung parenchyma (Clusters 1 and 4). The ordering of transcriptional laminae at the perinecrotic corona was heterogeneous between and within samples (Clusters 2, 9, 10, 15, and 16), but the bulk of the interior cellular layers consistently appeared as a leukocyte neighborhood with abundant aggregated epithelioid macrophages. At the most interior, we found perinecrotic clusters differentially enriched for multiple responses, including pathogen recognition, cytokine signaling, hypoxia, and metal stress, all known components of granuloma physiology (Fig. S3). An overlaying fibroid neighborhood (Clusters 3 and 6) with extracellular matrix (ECM) remodeling signatures was interspersed with lymphocytic infiltrate (Clusters 5, 7, and 8) expressing prominent humoral response elements.

### Paired single-cell transcriptomics of human pulmonary *M. tb* granulomas

**Fig 3:**
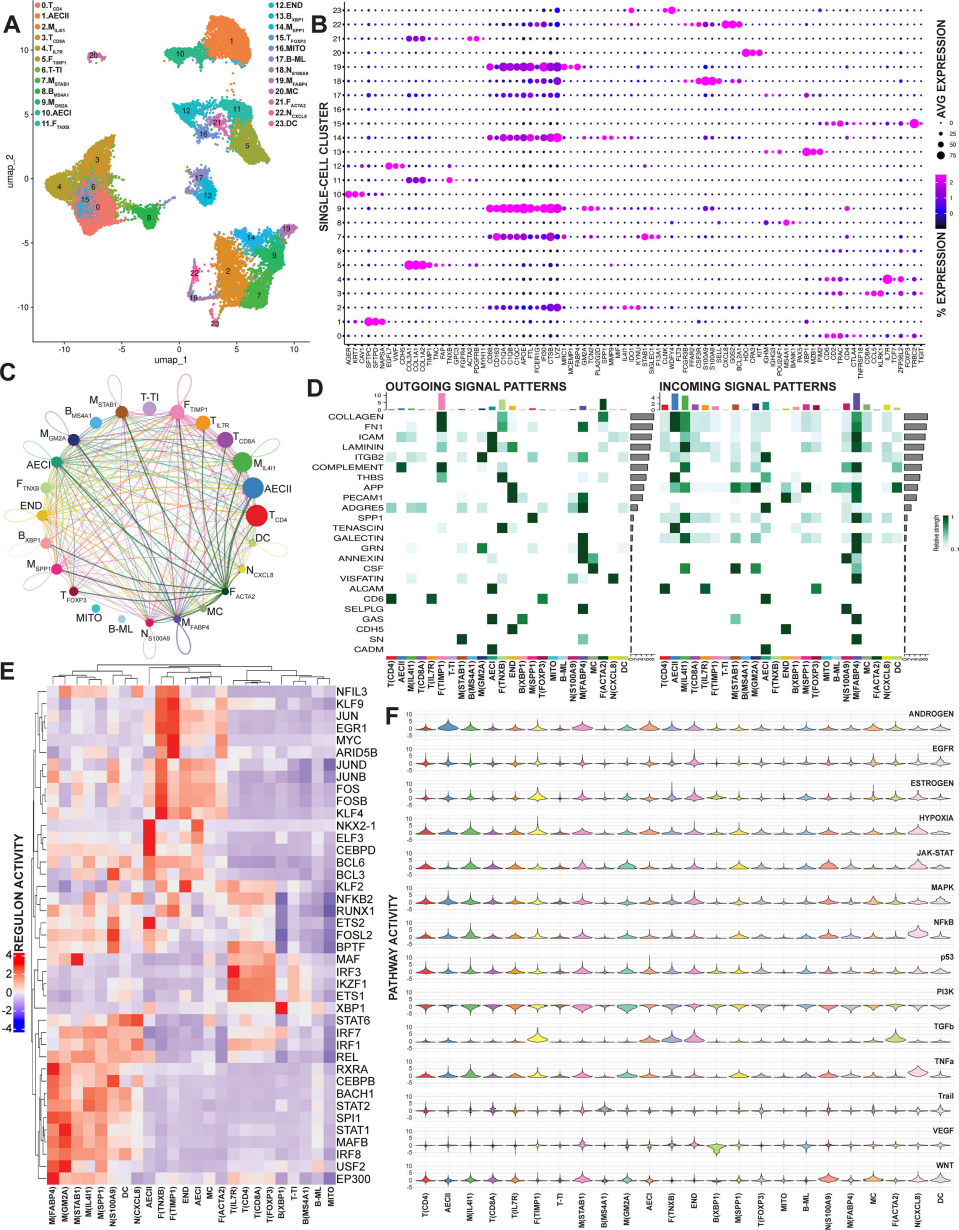
The combined single-cell transcriptomes of four pulmonary granuloma samples (P1, P2, P3a, and P3b) from three patients with TB. (**A**) A UMAP plot of annotated single-cell clusters generated by unsupervised clustering of integrated data from the four samples. (**B**) A bubble plot of the relative expression values for manually selected marker genes used for annotation across 24 normalized single-cell clusters. (**C**) Cell-cell communication inference from CellChat was used to generate a chord diagram representing the signaling network between single-cell clusters and (**D**) a heatmap plot showing the relative strength of the top outgoing and incoming signals across clusters. (**E**) SCENIC network inference was used to generate a heatmap plot of transcription factor regulon activity scaled by cell type. (**F**) A stacked violin plot of 14 pathway activity signatures across normalized single-cell clusters as determined by PROGENy inference. Pathway activity scores differed significantly across clusters for all 14 pathways (Kruskal-Wallis FDR-adjusted *P* < 0.0001).

Next, we evaluated gene expression in human *M. tb* granulomas by single-cell analysis. To focus more deeply on granulomas in pulmonary tissue, the predominant site of TB disease, we processed granuloma biopsy specimens resected from three patients with pulmonary TB (P1, P2, and P3). To increase the resolution of this analysis, and pair it to our spatial data, tissue processed for single-cell analysis from P1 and P2 was taken from the same biopsy samples in sections that were immediately adjacent to the sections used for Visium imaging. We used these closely paired dissociated cells to determine at a single-cell level the transcriptional signature and cellular heterogeneity in human *M. tb* granulomas and then comprehensively mapped these transcriptional profiles onto the spatial analysis using deconvolution algorithms. Subsequent clustering analysis of the combined data revealed 24 distinct cell populations, with cell-type-specific gene signatures (Fig. 3A; Fig. S4; Table S2). Cluster identity was assigned according to the expression of established cell-type specific transcripts (marker genes) (Fig. 3B) and evaluation of cluster-specific inferential pathway enrichment by gene set variation analysis (GSVA) (Fig. S5).

The most abundant cell populations in the granulomas we evaluated were leukocytes (Fig. S4B). We found large numbers of lymphocytes in these granulomas. There were three B cell (*IGHM*, *IGHG3*, and *POU2AF1*) subtypes: (i) B_MS4A1_ mature B cells (cluster 8: *MS4A1*, *BANK1*, *PAX5*), (ii) B_XBP1_ plasma B cells (cluster 13: *XBP1*, *MZB1*, *PIM2*), and (iii) B-ML, a distinct cluster of granuloma B cells that expressed elevated levels of both B cell and macrophage transcripts (cluster 17). Intriguingly, these macrophage-like B cells (29) had elevated levels of myeloid markers such as *LYZ*, and *ITGAX,* along with prominent expression of genes that were also induced across macrophage clusters, such as *CD74*, *APOE,* and *FTL*. Five T-cell (*CD6*, *CD2*, *TRAC*) subtypes populated these granulomas: (i) T_CD4_ helper T cells (cluster 0: *CD4*, *CTLA4*, *TNFRSF18*), (ii) T_CD8A_ cytotoxic T cells (cluster 3: *CD8A*, *CCL5,* and *KLRK1*), (iii) T_IL7R_ memory helper T cells (cluster 4: *IL7R*, *TCF7*, *ZFP36L2*), (iv) T_FOXP3_ regulatory T cells (cluster 15: *FOXP3*, *TRBC2*, *TIGIT*), and (v) T-TI, a cluster of transcriptionally inactive T cells that may be in a stressed, anergic, or exhausted state (cluster 6).

A diverse array of innate immune cells, including mast cells (MC) (cluster 20: *HDC*, *CPA3*, *KIT*), dendritic cells (DC) (cluster 23: *CLNK*, *WDFY4*, *FLT3*), neutrophils (*FCGR3B*, *FFAR2*, *CSF3R*), and macrophages (*CD68*, *CD163*, *C1QA*/*B*/*C*), was also present in these granulomas. There were two distinct neutrophil subtypes: (i) N_S100A9_ mature neutrophils with an elevated expression of granule components (cluster 18: *S100A9*, *S100A8*, *SELL*) and (ii) N_CXCL8_, an activated neutrophil population with a transcriptional signature (cluster 22: *CXCL8*, *G0S2*, *BCL2A1*) that is associated with pathogenic inflammation in other illnesses (30–32). We identified a macrophage population, M_FABP4_, with an expression profile consistent with alveolar macrophages (cluster 19: *FABP4*, *MRC1*, *MCEMP1*). Four granuloma-specific macrophage populations were also identified: (i) M_GM2A_ macrophages had an elevated lipid association expression profile (cluster 9: *GM2A*, *TCN2*, *CHIT1*), (ii) M_IL4I1_ macrophages were enriched with genes of the kynurenine pathway (cluster 2: *IL4I1*, *IDO1*, *KYNU*), (iii) M_STAB1_ macrophages had elevated expression of immunosuppressive receptors (cluster 7: *STAB1*, *SIGLEC1*, *F13A1*) similar to neuron-associated macrophages (33), and (iv) M_SPP1_ macrophages displayed a tissue remodeling signature (cluster 14: *SPP1*, *MMP9*, *MIF*).

Resident cell populations from local pulmonary tissue were also identified, including type I alveolar epithelial cells (AECI) (cluster 10: *AGER*, *KRT7*, *CAV1*), type II alveolar epithelial cells (AECII) (cluster 1: *SFTPC*, *SFTPD*, *NAPSA*), and endothelial cells (END) (cluster 12: *EGFL7*, *VWF*, *CDH5*). These tissue samples also contained three distinct populations of fibroblasts (*COL1A1*, *COL1A2*, *COL3A1*): (i) F_TNXB_ alveolar fibroblasts (cluster 11: *TNXB*, *GPC3*, *FGFR4*), (ii) F_ACTA2_ myofibroblasts (cluster 21: *ACTA2*, *PDGFRB*, *MYH11*), and (iii) F_TIMP1_, a distinct activated fibroblast population (cluster 5: *TIMP1*, *TNC*, *FAP*). MITO, a cluster highly enriched in mitochondrial transcripts, was assigned to necrotic cells (cluster 16). Cumulatively, these findings confirm that human *M. tb* granulomas and surrounding tissue consist of diverse cell types with gene expression patterns that are simultaneously specific to granulomas and broadly comparable to cellular differentiation states found in other pathologies.

### Single-cell interaction analysis

Having established the granuloma’s cellular constituency, we determined probable interactions between those populations. To do so, we used CellChat systematic analysis (34) to infer the dominant cell communication networks in our single-cell data set (Fig. 3C and D). ECM ligand-receptor interactions were a prominent feature of these granulomas (Fig. S6). Highly expressed fibroblast-derived ECM collagens, laminin, and fibronectin were likely recognized by receptors expressed in most of the cell populations we described. SDC1 and SDC4 receptors that were expressed by AECII cells in our tissue specimens likely recognized fibroblast collagens but also may have facilitated pneumocyte infection by *M. tb* (35, 36). In addition to ECM genes, F_TIMP1_ populations highly expressed complement C3, which can opsonize *M. tb* (37) and could have signaled to receptors on macrophage and neutrophil subsets. Among leukocyte populations, M_FABP4_ had the most predicted cell-cell interactions, with abundant production of both ligand and receptor signals. In comparison, the M_IL4I1_ cluster could receive many signals but transmitted relatively few. CD44 was the most prominent signaling hub in our granulomas. M_SPP1_ OPN and M_FABP4_ fibronectin were among the most prominent macrophage ECM ligands sensed by CD44, which likely also received signals from M_FABP4_-derived galectin-9 and multiple fibroblast collagens and laminins (Fig. S6). The interactions predicted between cell types in our analysis highlight a limited set of well-characterized receptor-ligand interactions that were prevalent in the data. However, these inferences should not rule out cell-cell interactions not included in these results.

### Single-cell population regulatory inference

Next, we determined the primary transcriptional regulators in these cell populations. We used the SCENIC workflow (38) to map gene regulatory networks, infer regulons, and predict which transcription factors were responsible for cluster-specific gene expression in our single-cell pulmonary granuloma data set (Fig. 3E). As expected, we found similar transcription factor activity between clusters with the same general cell-type designation. Shared regulons were particularly pronounced within fibroblast (KLF9, MYC, and EGR1), T cell (ETS1, IKZF1, and IRF3), and macrophage populations (IRF8, STAT1, and SPI1). Some non-macrophage populations, including F_TNXB_ (KLF4), F_TIMP1_ (ARID5B and MYC), B_XBP1_ (XBP1), N_CXCL8_ (STAT6), N_S100A9_ (FOSL2 and BCL3), and T_IL7R_ (IRF3), had unique transcription factor activity profiles.

Similar patterns of regulon activity between clusters within the general macrophage designation provided context for downstream analysis. M_FABP4_ cells had elevated activity by PPARG cistrome constituents RXRA and EP300 (39). PPARG was differentially expressed by M_FABP4_ (Table S2) and is a definitive hub of alveolar macrophage identity (40). M_STAB1_ cells had a uniquely high MAF activity, which is consistent with their elevated expression of IL10 (Table S2) and an immunosuppressive macrophage polarization state during *M. tb* infection (41–43). M_GM2A_ had elevated STAT1 activity, whereas M_IL4I1_ had elevated STAT2 activity, indicating activation by distinct classes of interferons or other inflammatory mediators. Interestingly, BACH1 activity was elevated in both M_SPP1_ and M_IL4I1_ and has been associated with increased susceptibility to ferroptosis during *M. tb* infection (44).

### Single-cell population pathway activity prediction

Finally, we inferred signaling activity in our single-cell populations using PROGENy pathway analysis (45, 46) (Fig. 3F). Some cell types showed notable pathway induction. Inflammatory pathway activation was a prominent feature of neutrophils and macrophages. Neutrophil and macrophage populations also had elevated JAK-STAT signaling, whereas VEGF pathway activation, a contributor to granuloma angiogenesis (47), was enhanced in endothelial cells. In addition, a TGFβ pathway signature was prominent in fibroblasts.

### Spatial distribution of single-cell populations

**Fig 4:**
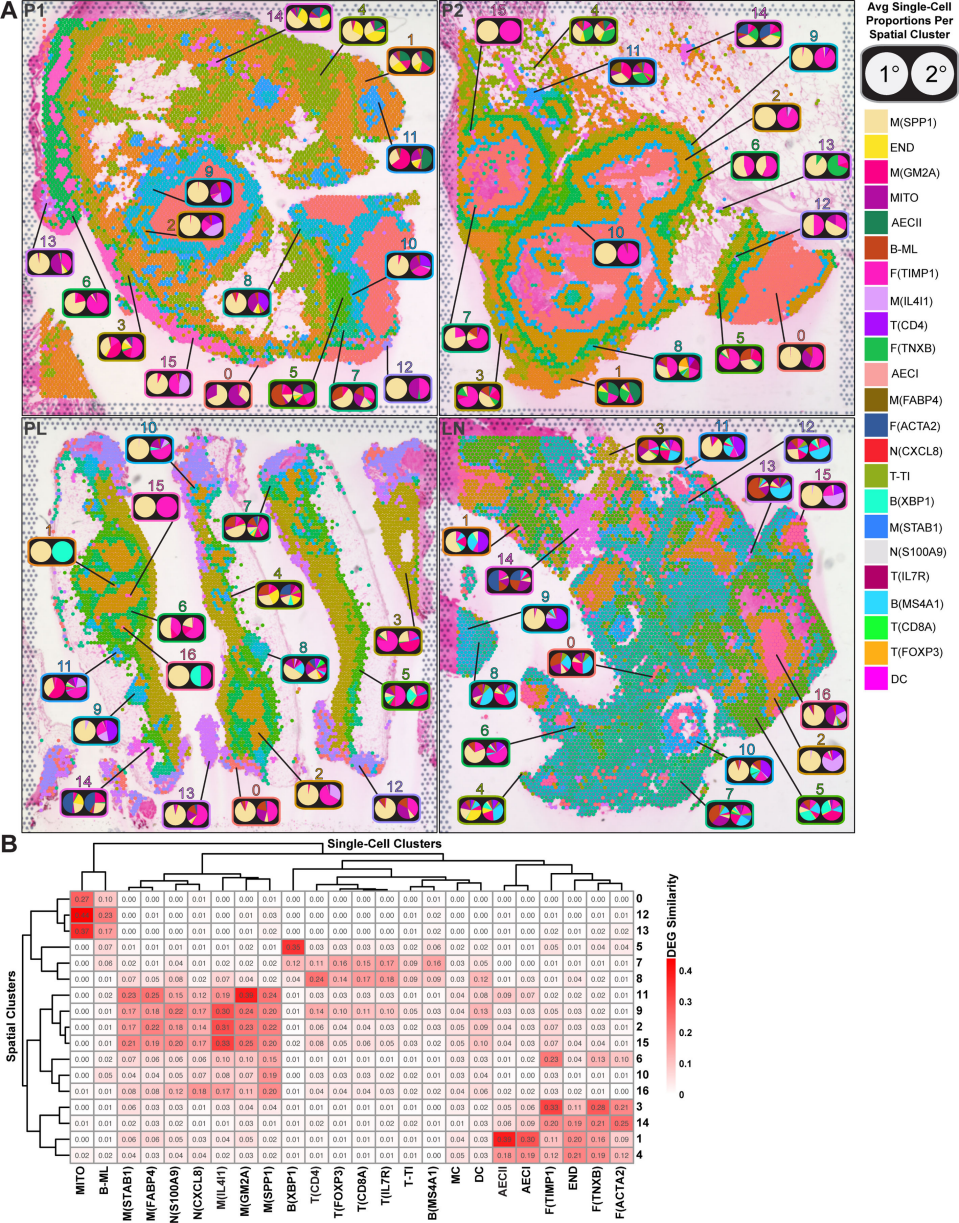
The deconvolution of spatial and single-cell clusters provides a blueprint of cell-type-specific regional occupancy in human *M. tb* granulomas. (**A**) RCTD decomposition estimates the fraction of primary-major (left) and secondary-minor (right) cell-type occupancy of each spot. Pie charts representing the average spot composition in each spatial cluster region were manually annotated onto Visium v2 spot maps. (**B**) A heatmap plot showing the similarity between spatial and single-cell clusters as determined by the Jaccard similarity index (where 0 is completely different and 1 is the same).

To identify location-specific niches for granuloma single-cell populations, we deconvolved the paired spatial and single-cell transcriptomes. Using the robust cell type decomposition (RCTD) methodology (48), we decomposed cell type mixtures of each spatial spot into doublets of major-primary (1°) and minor-secondary (2°) populations and then integrated the averages across our previously defined spatial clusters to produce sets of detailed architectural blueprints of the human *M. tb* granulomas we analyzed (Fig. 4A; Fig. S7). Similarity comparison of differentially expressed genes between single-cell and spatial clusters (Fig. 4B) supported those deconvolution results. Strikingly, we found that M_SPP1_ is almost always the major primary population in granuloma-associated spatial clusters and makes up a significant proportion of every spatial cluster in those lesions (Fig. 4A). This suggests that human *M. tb* granulomas are aggregates of M_SPP1_ that are infiltrated with and surrounded by accessory cell types. In that context, spatial laminae were generally populated by specific cell types, and the distribution of those populations was organized differently in the individual granulomas that we evaluated.

Consistent with lymphocytic infiltration, necrotic granulomas in P1 had abundant T_CD4_ within and surrounding a perinecrotic neighborhood that consisted of M_IL4I1_ interspersed with areas of N_CXCL8_ and F_TIMP1_. Outer regions were layered with M_GM2A_, B-ML, and F_TIMP1_. Peripheral satellite foci were organized mostly with central M_IL4I1_ and N_CXCL8_ surrounded by T_CD4_ beneath M_GM2A_ or F_TIMP1_ interspersed with M_GM2A_. Consistent with more advanced fibrotic pathology, P2 necrotic granulomas had robust populations of F_TIMP1_ throughout a thick intermediate myeloid neighborhood that contained sparse M_IL4I1_. F_TIMP1_ was present in higher proportions in a fibrotic neighborhood that encapsulated the myeloid neighborhood, which is easily distinguishable histologically (Fig. 1D). Significant populations of M_GM2A_ and B-ML in the outermost laminae were occasionally interspersed with T-cell populations. Noteworthy B-ML signatures were found within the necrotic core, which may be artifactual or represent lymphocytic invasion of the caseum. Cellular granulomas in PL had central F_TIMP1_ and M_IL4I1_ populations with a few T_CD4_-rich lesions and outlying tissue infiltrated with M_GM2A_ and B-cell populations. The periphery of some granulomas had areas with conspicuous M_STAB1_ populations. LN granulomas had abundant M_IL4I1_ within and surrounding areas of necrosis that at points had T_CD4_ and B-ML infiltration. The bulk of the surrounding tissue was comprised of B-cell populations in LN.

### Granuloma macrophages

**Fig 5:**
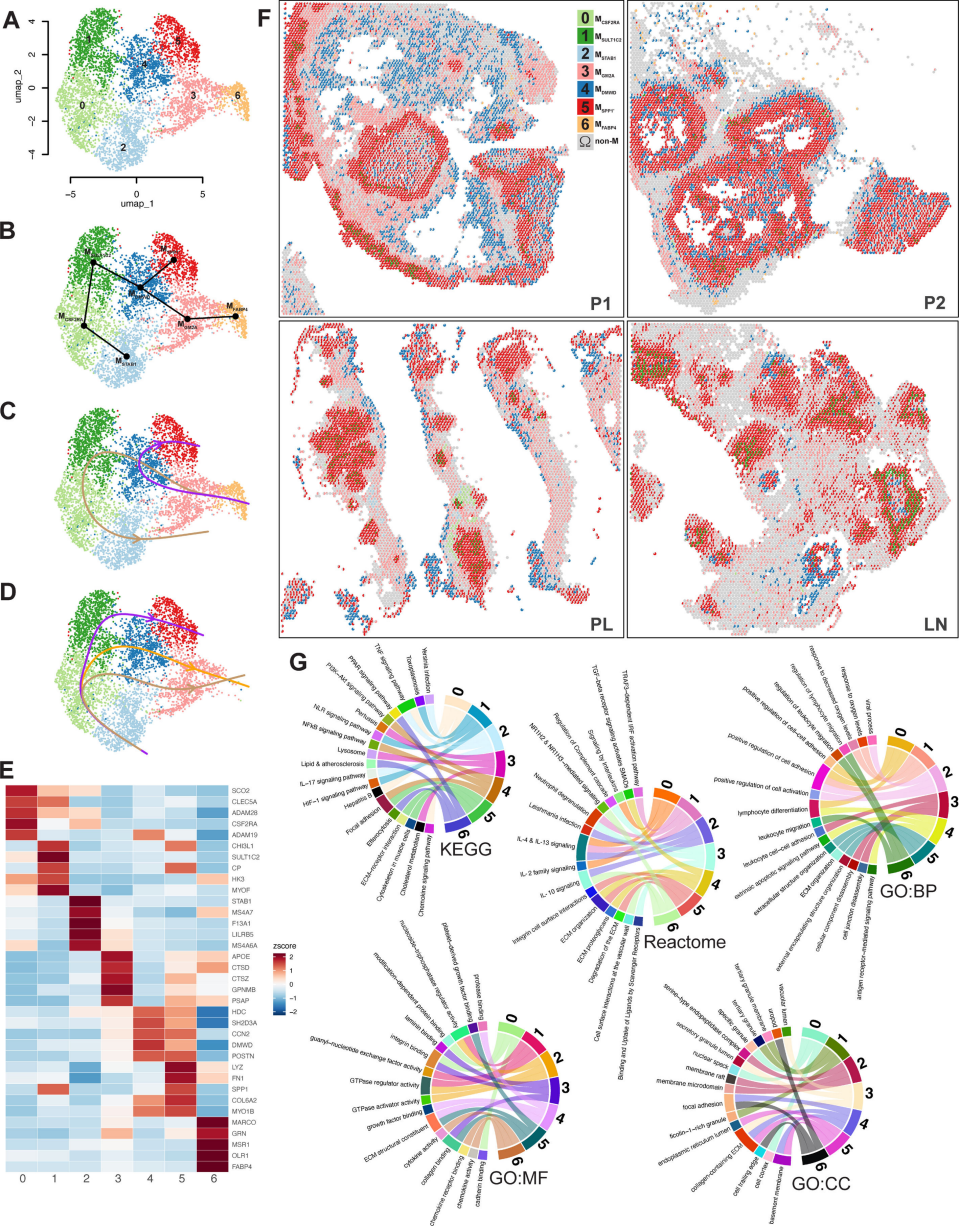
Granuloma macrophage diversity analysis. (**A**) A UMAP plot shows seven macrophage subclusters (0 = MCSF_CSF2RA_, 1 = MSULT_SULT1C2_, 2 = MSTAB_STAB1_, 3 = MGM_GM2A_, 4 = M_DMWD_, 5 = MSPP_SPP1_', and 6 = MFABP_FABP4_) from reanalysis of macrophage cells in the granuloma single-cell RNA-seq data. Slingshot analysis shows the connection of macrophage cluster nodes with a minimum spanning tree (**B**), and trajectory inference in the presence of the M_FABP4_ alveolar macrophage population (**C**) or its absence (**D**). (**E**) A heatmap plot of the top five differentially expressed genes in the seven macrophage subclusters. (**F**) Pie charts from RCTD deconvolution inference of the four Visium v2 spatial plots showing the relative occupancy of spots by the seven macrophage subtypes when they are the primary-major or secondary-minor populations (Cluster colors are matched to those in A, all other cell types in gray). (**G**) Chord plots of the top three pathways enriched in macrophage subsets by over-representation analysis of differentially expressed genes (adj. *P* < 5 × 10^−5^ and log₂FC > 0.5) using KEGG, Reactome, and Gene Ontogeny (Biological Process, Molecular Function, and Cellular Component) databases filtered using an adj. *P* < 5 × 10^−7^.

Having established the prevalence of M_SPP1_ populations spatially in the human TB granulomas that we assayed, we reanalyzed the macrophage supercluster in our single-cell experiment (Fig. 3A) by Slingshot lineage inference (49). M_FABP4_ (Cluster 6), M_GM2A_ (Cluster 3), and M_STAB1_ (Cluster 2) were mostly retained, but four new macrophage populations emerged (Fig. 5A). M_CSF2RA_ (Cluster 0) and M_SULT1C2_ (Cluster 1) were formed from the division of M_IL4I1_. M_DMWD_ (Cluster 4) and a new M_SPP1_' (Cluster 5) were formed primarily from the division of the original M_SPP1_. To understand how granuloma macrophage populations might change over time, we used Slingshot pseudotime inference (49) to predict granuloma macrophage lineage trajectories based on the seven subclusters (Fig. 5B). Granuloma macrophages were predicted to develop from M_FABP4_ alveolar macrophages, progress into the M_GM2A_ state and through the M_DMWD_ transition state either to the M_SPP1_' state or to the M_SULT1C2_ state then on into the M_CSF2RA_ state and finally the M_STAB1_ state (Fig. 5C). In the absence of alveolar macrophages as observed in PL and LN, Slingshot predicted alternative trajectories that began with M_STAB1_ and ended with either M_SPP1_' or M_GM2A_ (Fig. 5D). Although these predictions need to be confirmed by additional methods, they suggest macrophage plasticity that may underlie granuloma progression and heterogeneity. Individual macrophage clusters had distinct transcriptional profiles (Fig. 5E; Fig. S8A) that were used for cluster designation.

RCTD deconvolution of the new macrophage clusters revealed that M_SPP1_' dominated the granuloma, whereas its sister population M_DMWD_ resided in discrete tissue locations, consistent with it being responsible for the original M_SPP1_ identity in some spatial clusters. M_FABP4_ was exclusive to P1 and P2 in the outlying pulmonary tissue, whereas M_GM2A_ had extensive spatial distribution on the outer areas of granulomas consistent with spatial clusters 3 and 11 (Fig. 4A). The spatial profile of M_SULT1C2_ mostly overlapped with that of M_IL4I1_, whereas M_CSF2RA_ and M_STAB1_ had very limited distribution outside of areas peripheral to some granulomas in PL (Fig. 5F). Macrophage states had distinct pathway enrichment profiles consistent with diversification of functional identities (Fig. 5G; Table S3).

### Mycobacterium-induced macrophage OPN promotes granuloma formation

**Fig 6:**
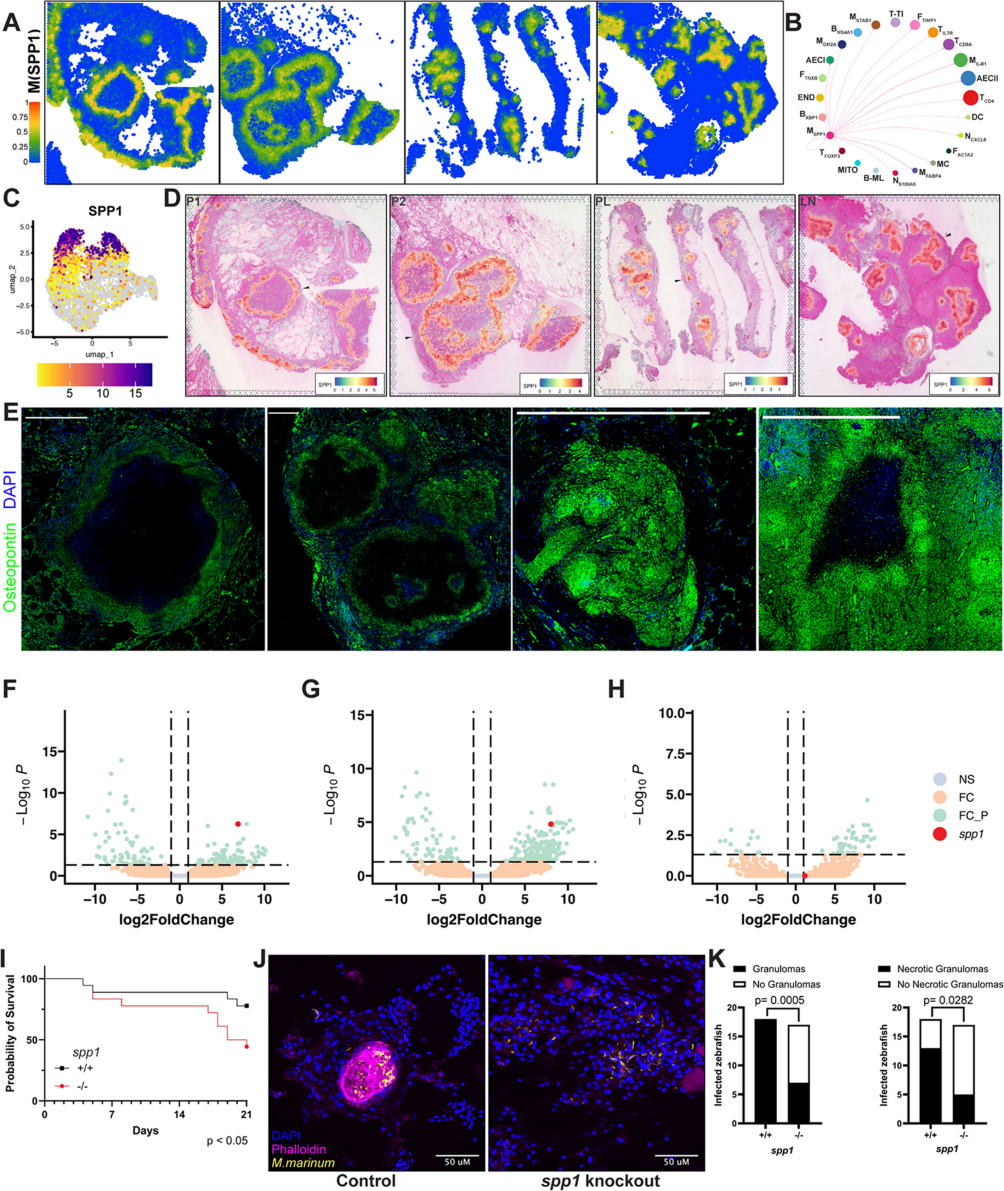
Determination of the role of macrophage *SPP1* expression in mycobacterial granulomas. (**A**) Spatial heatmap plots of the distribution of M_SPP1_ in human TB granulomas. (**B**) A chord diagram generated using CellChat inference that represents outgoing signaling from M_SPP1_ to other cell types. (**C**) A UMAP heatmap plot showing higher *SPP1* expression in macrophages of subclusters 1, 4, and 5 in the single-cell granuloma data set. (**D**) Spatial heatmap plots showing the location and relative intensity of *SPP1* expression in the spatial granuloma data set (black arrows indicate the location of IF-stained granulomas). (**E**) Representative photomicrographs of immunofluorescence staining for OPN in human *M. tb* granulomas in the same four biopsy specimens used in the spatial analysis (green = osteopontin, blue = nuclei). Volcano plots of bulk mRNA-seq in zebrafish show significance and magnitude of expression changes of differentially expressed genes, including *spp1* (red dot) in (**F**) infected macrophages vs uninfected macrophages from infected animals, (**G**) infected macrophages from infected animals vs uninfected macrophages from uninfected control animals or (**H**) uninfected macrophages from infected animals vs uninfected macrophages from uninfected control animals as determined by mRNA-Seq of flow-sorted red fluorescent macrophages obtained from larval zebrafish infected with green fluorescent *M. marinum* or uninfected (NS = non-significant values, FC = significant log_2_fold change and non-significant -log_10_ *P* values, FC_*P* = significant log_2_fold change and significant -log_10_ *P* values). (**I**) Kaplan-Meier curves of 3-week survival between control or *spp1*-knockout adult zebrafish infected with *M. marinum* (*P* value computed using the log-rank test). (**J**) Representative photomicrographs of tissue sections from 18 control or 17 *spp1*-knockout adult zebrafish infected with *M. marinum* showing a typical nidus of infection (yellow = *M. marinum*, magenta = actin, blue = nuclei). (**K**) Quantitation of the number of infected animals that had at least one granuloma or necrotic granuloma in tissue sections from J (*P* value computed using the chi-squared test with Yates correction).

Differential expression of *SPP1* defined a macrophage population that dominated the deconvolved spatial signature of the granulomas we analyzed (Fig. 6A). SPP1 was the major signal from M_SPP1_ to most other granuloma cell types via interaction with CD44 (Fig. 6B, Fig. 3D; Fig. S6) (50). *SPP1* was highly induced in multiple macrophage single-cell clusters (Fig. 6C; Fig. S8B) and was consistently elevated in the entire myeloid cuff in each of the granulomas we evaluated (Fig. 6D). To match the transcriptional findings to protein-level analysis, we stained for OPN protein and found that it was also robustly produced in the myeloid cuff of human *M. tb* granulomas (Fig. 6E). Therefore, we chose to assess the utility of our human *M. tb* granuloma data set as a pathway to discovery in TB pathogenesis by examining the role of OPN in mycobacterial granuloma formation.

We evaluated the role of osteopontin in granuloma formation using the larval and adult zebrafish-*M. marinum* models (15, 51). To determine the impact of mycobacterial infection on the transcriptional response of nascent granuloma macrophages, we conducted bulk RNA-seq on flow-sorted infected and uninfected macrophages isolated from infected larvae or uninfected controls with fluorescently labeled macrophages and differentially labeled bacteria. We used a low RNA input protocol developed by Zhou et al. (52) and generated a data set that enables comparison of paired infected and uninfected macrophages isolated from the same animals as well as from uninfected control animals (Fig. 6F through H). These classes of differentially regulated macrophage transcripts are included in Table S4.

We discovered that zebrafish *spp1*, the ortholog of human *SPP1* (53), was dramatically induced in infected macrophages (Fig. 6F and G). It was not induced in uninfected bystander macrophages from the same infected larvae (Fig. 6F) or expressed at high levels in uninfected controls (Fig. 6H). *spp1* was the most striking example of only a few genes that, at early infection timepoints, are cell autonomously upregulated in infected cells (Table S4).

To assess the functional role of *spp1* in mycobacterial granulomas, we tested a zebrafish *spp1* knockout mutant (54) in two separate models of *M. marinum* infection. Using larvae from the *spp1* knockout, we found no increase in mycobacterial burden during the initial stages of infection (Fig. S8C). To examine potential roles for *spp1* in more mature granulomas, we evaluated mycobacterial infection in adult zebrafish, which form more mature and complex caseating granulomas that have shared features with human granulomas (51). We found that during *M. marinum* infection in adults, *spp1* knockout animals displayed decreased survival over 3 weeks of infection as compared to controls (Fig. 6I). To examine whether *spp1* might be acting through alterations in granuloma organization or stability, we harvested infected *spp1* knockout and control adult zebrafish at 2 weeks post-infection, a timepoint when organized mature granulomas emerge consistently in wild-type animals. We observed a substantial defect in the formation of mature granulomas in *spp1*-deficient animals compared with *spp1*-expressing controls (Fig. 6J and K), suggesting an important conserved role for *spp1* in granuloma formation in response to pathogenic mycobacteria.

## DISCUSSION

Advances in RNA-sequencing technologies have enabled high-resolution spatial analyses of key cell types and transcripts that may underlie pathologic structures in disease. Here, we coupled Visium spatial technology with single-cell RNA expression analysis to produce a high-resolution view of human tuberculous granulomas and illustrated the key role of osteopontin-expressing macrophages in organizing these granulomas. Deconvolution analysis provided a set of blueprints of TB granuloma cellular infrastructure and highlighted the centrality of macrophage subtypes to granuloma organization (Fig. 4). After careful evaluation of macrophage identity (Fig. 5), we found that human tuberculous granulomas across patients and diverse *M. tuberculosis* strains are marked by organized aggregates of a subset of macrophages M_SPP1_', defined by their expression of OPN and other ECM components (Fig. 5F and G; Table S3). *SPP1* is prolifically expressed by the three populations of macrophages that inhabit the myeloid cuff (Fig. 6C and D) where OPN protein is likewise extremely abundant (Fig. 6E). To understand the functional significance of SPP1 in these macrophages, we performed a complementary set of experiments to examine *spp1*, the zebrafish homolog of human *SPP1*, in an *M. marinum* infection model. We showed that *spp1* expression was specifically induced in macrophages that had engulfed mycobacteria (Fig. 6F through H). Furthermore, *spp1* knockout animals had a defect in granuloma formation (Fig. 6J and K) that translated to reduced survival (Fig. 6I).

OPN is a pleiotropic matricellular protein with intracellular (iOPN) as well as secreted extracellular soluble and polymeric forms (sOPN) (55–57). It can serve as a macrophage-derived ECM component that has a wide range of effects, including but not limited to matrix rigidity, leukocyte attraction, migration, adhesion, and differentiation (58, 59). OPN is highly expressed by human macrophages in granulomas from multiple tissues across diverse disease states (27, 60, 61). Increased OPN production was previously described in human TB granulomas and *M. tb-*infected pulmonary macrophages (25). Clinical parameters of disease severity, including lesion size, are correlated with OPN plasma levels in TB (26, 62). However, *SPP1* induction inversely correlated with disease progression and bacterial dissemination during mycobacterial infection of immunocompromised patients (63). In mice, *Spp1* deletion causes granuloma defects in some non-mycobacterial disease states (64, 65) but does not impact protection during *M. tb* infection in the C57BL/6 mouse model (63), where organized granulomas do not form. Interestingly, mouse strains that form necrotic granulomas, such as C3HeB/FeJ, include a prominent *Spp1* pathogenesis-associated macrophage population that is absent in strains like C57BL/6 (66).

Consistent with a pathological wound healing response, ECM production is a central physiological process in TB granulomas. ECM expression by F_TIMP1_ dominates intercellular interactions and encapsulates the structure with a fibrotic cuff (Fig. 4A and 1D). In our analysis, M_SPP1_' is the most robust cell type in granulomas and is in close association with F_TIMP1_ in many regions. M_SPP1_' is polarized to an ECM expressing state, similar in many ways to a population of granuloma macrophages previously predicted *in silico* (67) as well as a number of macrophage subsets in other pathological conditions (68–73). These matrix macrophages are phagocytes that specifically deposit OPN, fibronectin, and various collagens along with matrix metalloprotease-9 and lysozyme, likely toward the construction of a provisional matrix that is a fundamental milieu of the granulomatous response to mycobacterial infection. Production of ECM by macrophages is emerging as a cell-type specialization with implications across both chronic and infectious diseases (72, 74, 75). In the context of tuberculosis, matrix macrophages likely establish a zone of recruitment for leukocytes and fibroblasts to promote pathological tissue remodeling and granuloma formation. They may also contribute to bacterial control in granulomas by promoting granuloma fibocalcification (11) or with concurrent production of lysozyme (Fig. 5E), which could function to trap and kill large numbers of extracellular bacteria (76).

Surrounding and among the matrix macrophage aggregates, which formed the bulk of the granulomas, we evaluated heterogeneous collections of stromal and immune cells. Interior populations of note included hypoxic response-enriched macrophages as well as infiltrating T cells and neutrophils. Extensive populations of fibroblasts occupied the interior and extended out to an encapsulating fibrotic neighborhood around some lesions. Abundant lipid-laden macrophages and transition state macrophages were present at the granuloma’s margins and throughout the surrounding tissue. Frequently interspersed around the outer orbit of granulomas were B-cell-enriched lymphocytic foci (77), mainly composed of a population of B cells with macrophage characteristics that bear some resemblance to atypical granuloma B cells (78). Other cell types were represented in various proportions within samples and depending on tissue type.

By pairing cell dissociation from archived FFPE clinical samples with spatial analysis of adjacent matched sections, we were able to place individual cell subpopulations within 34 tuberculous granulomas in context. We identified new cell subsets that reflect the unique trajectory of mycobacterial infections as well as the diverse microenvironments that exist within granulomas. In addition to the discovery of a novel granuloma matrix macrophage population in human *M. tb* granulomas, we observed granuloma cell types and/or architectural strata that were consistent with established paradigms in TB pathogenesis including but not limited to immune tolerance (79), type 2 immunity (18), glycolytic metabolism (80, 81), granuloma hypoxia (82, 83), macrophage lipid metabolism (84, 85), and metal-dependent oxidative stress (44, 86, 87). Our findings overlap with ongoing spatial and/or single-cell transcriptomic analyses of human or non-human primate granulomas from our own and other groups (88–92) as well as detailed spatial profiling of specific marker genes (77, 93, 94).

Analysis of human clinical tissues provides a unique view into granuloma identity and evolution. These samples were identified agnostically in apparently immunocompetent patients. Conserved spatial and transcriptional programs suggest that many of these features are core features of tuberculous granulomas and do not represent outlier states. All the patients had active, culture-confirmed tuberculosis, and the granulomas were obtained from biopsies at sites of clinically active disease, so it is possible that the granulomas captured here represent ineffective host immune responses that differ in some aspect(s) from granulomas that effectively contain infection. We identified cell populations that are well conserved across species and consistent with descriptions from single-cell studies of granulomas from mycobacterial infections of zebrafish and non-human primates (17, 18, 91), as well as MIBI-TOF-based analysis of human granulomas (93). The spatial analysis described here, coupled to single-cell analysis on paired human sections, enabled spatial resolution of where these populations lie relative to the necrotic core and functional testing of a particular subpopulation of macrophages that rose to prominence in our analysis. Other populations that have not been well explored include a necrotic core adjacent neutrophil population N_CXCL8_ and a macrophage-like B-cell population B-ML.

Neutrophils are associated with susceptibility to mycobacterial infection in multiple animal models (95–97). Granuloma-associated neutrophils occupy the edges of necrotic cores in human and macaque TB (Fig. S7; P1) (96, 98). Analysis of single-cell data obtained from the blood of human patients identified neutrophils with transcriptional signatures similar to N_CXCL8_ as predictors of disease severity in sepsis (30), COVID-19 (31), and COPD (32). Parsing single-cell data from experimental *M. tb* infection of macaques revealed a population of granuloma neutrophils that share a similar transcriptional profile in that model system (17, 95). The N_CXCL8_ transcriptional signature is enriched in areas of granuloma P1 surrounding the necrotic core where populations of neutrophils are present and, in some places, have an apoptotic histological presentation (Fig. S7 and Fig. 1D). This transcriptional signature may represent a neutrophil cell death state indicative of stressed neutrophils encountering a necrotic microenvironment and signaling for additional neutrophil influx via IL-8.

Deposits of B-cell-rich tertiary lymphoid tissue are an established component of the granulomatous response to *M. tb* (77, 99). B-ML was the dominant B-cell signature within regions of tertiary lymphoid tissue around our human TB granulomas (Fig. S7). Furthermore, some locations within the necrotic core shared that signature, which could be artifactual, but could also indicate that this population carries out sorties into necrotic cores. B cells with co-expression of macrophage markers have been observed in and around the lesions of other diseases (29, 100). The implications of B-cell adoption of a phagocytic cell’s expression profile in granulomas remain unexplored as an aspect of TB pathogenesis.

We envision this work as a stepping stone to generate testable hypotheses related to functional roles of granuloma-related pathways in tuberculosis and even other granuloma-associated diseases like sarcoidosis. Here we used *SPP1* as an example of the power of these data to provide insights into the granulomatous response to *M. tb*. However, the robust data set generated here provides ample fodder for exploration and elucidation of critical questions in granuloma biology that are at the center of TB pathogenesis. We anticipate that these data will benefit investigations focused on factors that influence granuloma formation and maturation, as well as studies to identify new host-directed drug targets. Understanding the spatial organization of human granulomas may also facilitate the development and testing of more accurate *in silico* and *in vitro* granuloma models. Finally, this work uncovers macrophage-matrix synergism that may play an important role in tuberculous granuloma formation, progression, and resolution.

## MATERIALS AND METHODS

### Human patient specimens

A retrospective review of patients presenting to the Duke University Health System (DUHS) was conducted for pathology specimens reported to contain granulomas upon pathologic examination. Cases with available tissue specimens with positive cultures for *Mycobacterium tuberculosis* were evaluated for inclusion in the study. Tissue specimens were requested from the Duke University BioRepository & Precision Pathology Center (BRPC). Formalin-fixed paraffin-embedded (FFPE) sections were cut by the BRPC Research Histology Laboratory.

### Pathological examination

The presence of TB granulomas was confirmed by a board-certified anatomic pathologist following evaluation of H&E-stained sections for each sample included in this study.

### Preparation and analysis of spatial and single-cell samples

10× Genomics Visium V2 assays were carried out following 10× Genomics protocol CG000520-Rev B. FFPE tissues were sectioned to 5 μm thickness using a microtome and placed on a SuperFrost Plus slide (Fisher Scientific). The slides were then incubated at 42°C for 3 hours to ensure proper drying and stored at room temperature in a desiccator for up to 2 weeks before processing. Slides were stained for H&E and imaged at 40× magnification on a Zeiss Axioscan Z1 slide scanner. Tissues were then destained and decrosslinked to make RNAs accessible to downstream processing. Human whole transcriptome probe panels were added to the tissue, enabling hybridization of each probe pair across the transcriptome. Slides were processed through probe ligation and prepared for analyte transfer. Tissue slides and Visium CytAssist Spatial Gene Expression v2 Slides were then loaded into the Visium CytAssist instrument, where they were brought into proximity with one another. Gene expression probes were released from the tissue upon CytAssist Enabled RNA Digestion & Tissue Removal, enabling capture by the spatially barcoded oligonucleotides present on the Visium slide surface. The Visium CytAssist Spatial Gene Expression v2 slides were removed from the Visium CytAssist for downstream library preparation. Barcoded products were cleaved from the slide surface and amplified. Gene expression libraries were generated from each tissue section and sequenced on an Illumina NovaSeq 6000. Spatial barcodes were used to associate the sequencing reads back to the tissue section images for spatial mapping of gene expression.

For single-cell assays, 2–4 25 µM FFPE tissue sections of human TB granuloma biopsy specimens were dissociated into single cells and processed following the 10× Genomics protocol number CG000784. Nuclei and cells were isolated from FFPE tissue blocks using the 10× Genomics Sample Preparation from FFPE Tissue Sections protocol. Briefly, FFPE scrolls were deparaffinized through serial incubations in xylenes, ethanol, nuclease-free water, and PBS. Deparaffinized sections were then pestle dissociated into nuclei and cells using Dissociation Enzyme mix prepared with Liberase enzyme and media and incubation at 37°C for 45 minutes followed by aspiration through a 23G needle to improve recovery, filtration through a 30 µm Pre-Separation filter (Miltenyi Biotec, Bergisch Gladbach, North Rhine-Westphalia, Germany), washing with chilled PBS, and resuspension in tissue resuspension buffer. Isolated nuclei and cells were then counted on a Cellometer Automated Cell Counter (Revvity, Waltham, MA) using AOPI fluorescent dye.

Next, the 10× Genomics Fixed RNA Profiling Reagent Kits protocol was followed. First, fixed samples were centrifuged at 850 rcf for 5 min at 4°C, supernatant was removed, and resuspended in pre-warmed Hyb Mix at the concentration indicated as appropriate for the sample type. 20 µL of human WTA Probes BC001 was then added to each sample in Hyb Mix and incubated overnight at 42°C. After overnight incubation, samples were washed three times with post-Hyb Wash Buffer and then resuspended in chilled post-Hyb Wash Buffer and passed through a 30 µM filter Pre-Separation filter (Miltenyi Biotec, Bergisch Gladbach, North Rhine-Westphalia, Germany) and counted on a Cellometer to determine concentration for single-cell gel bead in emulsion (GEM) generation. Cells were stored according to long-term storage protocol with 50 µL of Enhancer and 137.5 μL of 50% glycerol. Probe barcoded samples, combined with a master mix containing reverse transcription (RT) reagents, were loaded onto the microfluidics chip, together with gel beads carrying the Illumina TruSeq Read 1 sequencing primer (Illumina, San Diego, CA), a 16 bp 10× barcode, a 12 bp unique molecular identifier (UMI) and a poly-dT primer, and oil for the emulsion reaction to generate nanoliter-scale gel beads in emulsion (GEMs). The 10× Genomics Chromium X instrument uses the microfluidics in the chip to partition the nuclei into nanoliter-scale gel beads in emulsion (GEMs) within which the RT reaction occurs; all cDNAs within a GEM, created from a single cell/nucleus, share a common barcode. After the RT reaction, the GEMs were broken, full-length cDNAs were cleaned with Silane Dynabeads, and then amplified via polymerase chain reaction (PCR) and purified using SPRIselect (Beckman Coulter, Brea, CA) bead size selection. Resulting cDNAs were assayed using a 4200 TapeStation System High-Sensitivity D5000 ScreenTape and reagents (Agilent, Santa Clara, CA) for qualitative and quantitative analyses.

Enzymatic fragmentation and size selection were used to optimize the cDNA amplicon size for the sequencing library preparation, in which Illumina P5 and P7 sequences, a sample index, and TruSeq read 2 primer sequence were added through end repair, A-tailing, and adaptor ligation PCR. The final libraries contain P5 and P7 primers used in Illumina bridge amplification. Following additional bead purification, libraries were assayed for quality using 4200 TapeStation System HSD1000 ScreenTape and reagents, then quantified, and checked for successful adapter ligation with the KAPA Library Quantification Kit (Roche, Indianapolis, IN) on the Applied Biosystems QuantStudio 5 (Thermo Fisher Scientific, Waltham, MA). Sequences were generated using paired-end sequencing (one end to generate cell-specific, barcoded sequence and the other to generate a sequence of the expressed poly-A-tailed mRNA). These libraries were sequenced on an Illumina NovaSeq 6000.

### Immunofluorescence of human tissue

FFPE tissue sections of human TB granuloma biopsy specimens were processed and stained as previously described (5). This study used rabbit anti-human osteopontin polyclonal antibody (#NBP1-89952, Novus Biologicals) diluted 1:200 or corresponding concentrations of rabbit IgG control (#10,500C, Invitrogen). Secondary staining was conducted using donkey anti-rabbit Alexa Fluor 555 (#A-31572, Invitrogen) diluted 1:500. Slides were mounted with DAPI Fluoromount-G (#0100-20, SouthernBiotech) and imaged on an ECHO Revolution epifluorescence microscope.

### Zebrafish lines

*spp1* mutant zebrafish contained a four-base-pair deletion in exon 7 on an EK background (54). Adult EK wild-type zebrafish were generously provided by John Rawls. Adult zebrafish heterozygous for *spp1*Δ4 were generated by outcross with lab stock AB adults then in-crossed to generate *spp1*Δ4 and control larvae. Transgenic zebrafish line *Tg(mfap4:tdTomato-CAAX)^xt6^* on an AB background were incrossed to generate larvae with macrophage-specific fluorescence (101).

### Mycobacterial single-cell stocks

Single-cell aliquots of M strain *Mycobacterium marinum* (#BAA-535, ATCC) expressing either mCeruleanfluorescent or Wasabi-fluorescent protein and hygromycin resistance under the *msp12* promoter (47) were prepared as previously described (102). Briefly, *M. marinum* was grown to an OD_600_ between 0.7 and 0.9 in 7H9 media (Difco #271310) with 10% OADC (#M0678, Sigma-Aldrich), 0.05% Tween-80 (#P1754, Sigma-Aldrich), and 50 µg/mL hygromycin B (#10687010, Invitrogen). Single-cell suspensions were made by repeatedly passing bacteria through a 27G syringe followed by a 5 µm filter. Bacteria were evaluated for single-cell status and quantified by fluorescence microscopy, then aliquoted and frozen at −80°C. Single-cell stocks were thawed at room temperature and diluted prior to infection.

### Larval zebrafish infection

Larvae were maintained at 28.5°C in E3 medium supplemented with 1-phenyl-2-thiourea (#P7629, Sigma-Aldrich) at a final concentration of 45 µg/mL to prevent melanization. Two days post-fertilization, larvae were infected with approximately 100 fluorescent-expressing *M. marinum* and assayed for mycobacterial burden as previously described (15). Briefly, an inoculum of *M. marinum* single-cell solution diluted in 7H9 with 0.5% phenol red indicator (#0290, Sigma) was loaded into a pulled borosilicate needle (#BF100-58-10, Sutter Instruments) broken approximately 8 mm from the tip. Larvae were anesthetized in tricaine 160 mg/mL (#MS-222, Syndel) and microinjected into the caudal vein with the inoculum using a FemtoJet injector (Eppendorf). At 4 days post-infection (dpi), larvae were anesthetized with tricaine and arrayed on a microscope slide. *M. marinum* fluorescence within larvae was captured using a Zeiss Observer Z1 microscope with a 2.5× objective. Bacterial burden by fluorescence was calculated using ImageJ as previously described (103). Image analysis was blinded to genotype during measurement.

### Zebrafish larval cell sorting and RNA-sequencing

For each biological replicate, ~500 2 dpf *Tg(mfap4:tdTomato-CAAX)^xt6^* zebrafish larvae were infected as above using wasabi-expressing *M. marinum; ~*200 2 dpf, *Tg(mfap4:tdTomato-CAAX)^xt6^* zebrafish larvae were reserved in parallel as uninfected controls with red-fluorescent macrophages; and lab stock wild-type AB (non-fluorescent) 2dpf were reserved as controls with no fluorescent markers. At 2 dpi, larvae from each group were anesthetized with tricaine and transferred into 1.5 mL tubes (~100 larvae per tube). Excess fish water was removed, replaced with 1 mL calcium-free deyolking buffer (55 mM NaCl, 1.8 mM KCl, 1.25 mM NaHCO_3_), tubes were inverted a few times. Following a 5 min incubation at RT, tubes were spun at 310 × *g* for 1 min to coalesce larvae at the tube bottoms. Deyolking buffer was removed and replaced with 1 mL of 0.25% trypsin/EDTA (Gibco/Invitrogen cat# 25200056). Larvae were physically dissociated by pipetting the larval contents ~5 times with a p1000 pipette every 10 min, and then the tubes were incubated at 28°C in between pipetting. After ~40 min, tubes were pipetted every 5 min and visually reviewed under a dissecting microscope to assess the progress of digestion. The infected larvae tended to digest more quickly than the controls, so digestion times ranged from ~50 to 60 min. Once digestion was complete (as assessed by visual inspection through a dissecting microscope), tubes were spun for 5 min at 660 × *g* at RT. All remaining steps were carried out on ice. The supernatant was removed and replaced with 1 mL ice-chilled PBS + 5% FCS (FBS had been heat-inactivated for 30 min at 56°C), and contents were pipetted up and down to mix, and then spun for 7 min at 200 × *g* at 4°C. Supernatant was discarded and cells were re-suspended in resuspension buffer 700 µL PBS + Mg + Ca (Lonza cat# 17-513F) + 2% FCS on ice. Then the cell suspension was pipetted onto a 40 µm filter to obtain single-cell suspensions (nylon cell strainer Falcon cat# 352340), and then an extra 100–200 µL resuspension buffer was added to help cells pass through the filter. All filtered re-suspensions from the same genotype were combined and then separated into 2 mL tubes to concentrate cells by spinning for 7 min at 1,450 rpm at 4°C. Supernatant was removed, and then all cell pellets of the same genotype were combined into 1 mL of re-suspension buffer. To each tube, 7-aminoactinomycin (7AAD; Sigma A9400, a dead cell marker) was added to a final concentration of 5 µg/mL, and DNase I (Sigma D4513) was added to a final concentration of 10 µg/mL. Contents were transferred to di-H_2_O rinsed FACS tubes (12 × 75 mm polypropylene 5 mL Falcon (VWR cat# 60819-794) for cell sorting. 5-cell collection tubes were prepared so that the sorted cells were directly stored in a solution specifically designed for RNA extractions (each collection tube contained 450 µL RLT Buffer (Qiagen micro RNeasy kit) and 4.5 µL beta-mercaptoethanol). Cell sorting was performed by the Duke Cancer Institute Flow Cytometry Shared Resource using either the Becton-Dickinson DiVa or Becton-Dickinson Astrios cell sorters. The sorting gates for FACS were established based on forward scatter and side scatter plots to capture cell populations based on expected morphology, and then gates were established to positively or negatively select populations based on fluorescence (using cells from AB, infected tdTomato, and uninfected tdTomato larvae to establish the gates). Cell population 1 was negative for td-Tomato and negative for 7AAD from uninfected td-Tomato larvae (Control −/−). Cell population 2 was positive for td-Tomato and negative for 7AAD from uninfected td-Tomato larvae (Control Red +). Cell population 3 was negative for td-Tomato and negative for 7AAD from infected td-Tomato larvae (Infected −/−). Cell population 4 was positive for td-Tomato and negative for 7AAD from infected td-Tomato larvae (Infected Red +). Cell population 5 was positive for td-Tomato, positive for wasabi, and negative for 7AAD from infected td-Tomato larvae (Infected Red +/green +). The population of double-positive cells collected was on the order of 1,500–2,500 cells. The population of other cell populations ranged from 20 to 100,000 and capped at 100,000. After cell sorting, collection tubes were stored at −80°C until ready for RNA extraction. RNA extraction was carried out using the Qiagen micro RNeasy kit and manufacturer’s instructions were followed, with the following exceptions: for the double-positive fluorescent cell group (with less than 2,000 cells) we added 5 µl of 4 ng/µL bacterial ribosomal RNA 16S/23S as a carrier (Roche cat# 10206938001); precipitated RNA at the final step with Ambion GlycoBlue (blue dye covalently linked to glycogen), 1× volume of 3 M Na acetate (pH 5.5), and 2.5× volume of 100% ethanol; and then resuspended the RNA pellet in 8 µL of molecular grade H_2_O. We followed the single-cell RT-PCR method for cDNA synthesis and subsequent PCR amplification (52). In brief, the equivalent RNA volume was calculated for ~500 cells per sorted population and used to generate cDNA with Anchor T primers, followed by primer removal, and then incubation with TdT and RNase H for the addition of poly(A) tails and subsequent PCR with Anchor T primers. The number of amplification cycles required was visually assessed by running a small amount of the PCR reaction at different cycle intervals. We also followed a previously described Illumina Sequencing Library Preparation method (52), whereby the amplified cDNA was purified with magnetic beads (Agencourt AMPure XP), and 50 ng of DNA was tagmentated using the Nextera DNA sample preparation kits (Illumina Inc). Sequencing was carried out by the Duke Sequencing and Genomic Technologies Shared Resource, using the Illumina HiSeq system with a 50 bp read length.

### Adult zebrafish infection and survival

Equal numbers of female and male adult zebrafish were anesthetized with 120 mg/L Tricaine. Single-cell aliquots of *M. marinum* were diluted in PBS to produce an inoculum with 350 fluorescent bacteria in 10 µL. Zebrafish were injected intraperitoneally with 10 µL of the inoculum using a 27G syringe (#08290-3284-38, BD). Infected zebrafish were recovered and maintained in spawning tanks at 28.5°C with daily health monitoring, feedings, and water exchanges.

### Preparation and immunofluorescence of zebrafish sections

Infected adult zebrafish were euthanized by Tricaine overdose. The torso was resected and then processed as previously described (104). Briefly, tissue was fixed with CLARITY hydrogel solution (4% paraformaldehyde, 4% acrylamide, 0.05% bis acrylamide, and 0.0025 g/mL VA-044) in 15 mL conical tubes for 3 days with gentle rocking at 4°C. A layer of mineral oil was added to prevent evaporation, and samples were placed into a 37°C water bath for 3 hours to polymerize. Samples were extracted from polymerized hydrogel and incubated with nutation for successive 24 h incubations in 10%, 20%, and then 30% sucrose/PBS solutions at room temperature. Tissue samples were then suspended in Neg-50 (#6502, epredia) and frozen at −80°C for at least 24 hours before cryo-sectioning at 10 µm and transferred to pre-cleaned slides (#15-188.48, Fisher), then stored at −80°C for at least 24 hours. Tissue sections were incubated in phalloidin Alexa Fluor 647 (# A22287, Invitrogen) during blocking and mounted using DAPI Fluoromount-G (#0100-20, SouthernBiotech) and cured for 24 hours before imaging on a spinning disk confocal microscope as previously described (5).

### Spatial snRNA-seq and scRNA-seq analysis

Raw BCL files were demultiplexed, and then short reads from spatial snRNA-seq were mapped to the human reference genome (GRCh38) using 10× Genomics Space Ranger version (2.0.1) with default parameters. The short reads from single-cell RNA-seq were mapped to the human genome (GRCh38) using 10× Genomics Cell Ranger (version 7.0). The filtered count matrix was retained for further analysis. Seurat (version 5.2.1) (105) was used for single-cell and spatial data processing and clustering, with initial clustering and visualization of spatial data conducted using 10× Genomics Loupe Browser (version 8). Cells with gene counts greater than 9,000 or less than 300 and a mitochondrial percentage greater than 20% were filtered out. CellChat (version 1.6.1) (34) was used for cell-cell communication inference, and SCENIC (version 1.3.1) (38) regulon analysis was completely unsupervised using the RcisTarget (version 1.24.0). Gene ontology and pathway enrichment analysis were performed using ClusterProfiler (version 4.15.1.1) (106). Overrepresentation analysis was visualized using chord diagrams generated by circlize (version 0.4.16) (107). Pathway activity scoring was performed either using GSVA (version 2.0.5) (108) with top variable pathways identified by median absolute deviation and visualized using pheatmap (version 1.0.12) or using PROGENy (version 1.28.0) (45, 46) and visualized using ggplot2 (version 3.5.1). Deconvolution analysis was performed using the RCTD algorithm via spacexr (version 2.1.1) (48). Pseudotime trajectory inference was performed using slingshot (version 2.14) (49).

### Bulk mRNA-seq analysis for zebrafish

Single-end short reads were mapped to the zebrafish genome (GRCz11) using Salmon (version 1.4.0) (109). Differential gene expression of zebrafish bulk mRNA-Seq data was conducted using DESeq2 (version 1.4.6) (110). Other hypothesis tests and visualization of zebrafish data were performed using Prism (version 10) GraphPad software.

### Statistical analysis

Hypothesis testing of human data was performed using Posit PBC RStudio statistical software (version 4.4.3). Statistical tests used for hypothesis testing are noted in the figure legends. Jaccard similarity coefficient between cluster A and B was calculated using the formula J(A, B) = |A∩B|/|A∪B|. Granuloma comparisons were tested using the chi-squared test with Yates’s continuity correction. Kaplan-Meier survival analysis was tested with the log-rank test. Larval burden analysis was tested by an unpaired Student’s *t*-test.

## Data Availability

Zebrafish RNA-seq, human spatial transcriptomics, and human single-cell RNA-seq are available at Gene Expression Omnibus (GEO) under accession numbers GSE296119, GSE296400, and GSE296399. The code used in the preparation of this paper is available at https://github.com/lw157/TBGranuloma. The paper and supplemental files include all other relevant data; additional raw data are available from the authors.

## AFTER EPUB

*[This article was published on 7 August 2025 without Acknowledgments. The Acknowledgments were added in the current version, posted on 12 August 2025.]*

## SUPPLEMENTAL MATERIAL

Supplemental Figures, part I mbio.01559-25-s0001.pdf Figures S1 to S5.

Supplemental Figures, part II mbio.01559-25-s0002.pdf Figures S6 to S8.

Table S1 mbio.01559-25-s0003.xlsx Differentially expressed genes unique to each of the 17 regions in TB granuloma tissue biopsy specimens P1, P2, PL, and LN that have a common transcriptional signature between samples, as determined by 10x Genomics Visium v2 spatial mRNA sequencing.

Table S2 mbio.01559-25-s0004.xlsx Normalized average expression values and differentially expressed genes for each of the 24 clusters identified in TB granuloma tissue biopsy specimens.

Table S3 mbio.01559-25-s0005.xlsx Differentially expressed genes unique to each of the seven macrophage populations in TB granuloma tissue biopsy specimens P1, P2, P3a, and P3b.

Table S4a mbio.01559-25-s0006.xlsx Normalized average expression values for infection conditions between macrophages that were infected (IMac) or uninfected (UMac) and were obtained from larvae infected with green fluorescent *M. marinum* (IL) or control uninfected larvae (UL), as determined by Illumina bulk mRNA sequencing of flow-sorted red fluorescent macrophages.

Table S4b mbio.01559-25-s0007.xlsx Continuation of Table S4.

ASM does not own the copyrights to Supplemental Material that may be linked to, or accessed through, an article. The authors have granted ASM a non-exclusive, world-wide license to publish the Supplemental Material files. Please contact the corresponding author directly for reuse.
