# Supplementary material for: Paired single-cell and spatial transcriptional profiling reveals a central osteopontin macrophage response mediating tuberculous granuloma formation
Source: mBio. 2025 Aug 7;16(9):e01559-25. doi: 10.1128/mbio.01559-25 (PMC12421895; doi:10.1128/mbio.01559-25)
Supplement: Supplemental Figures, part I — Figures S1 to S5. [file mbio.01559-25-s0001.pdf]

SUPPLEMENTARY FIGURES

Paired single-cell and spatial transcriptional profiling reveals a central osteopontin macrophage response mediating tuberculous granuloma formation in humans and zebrafish

Charlie J. Pyle[a,b,#], Liuyang Wang[a], Rebecca W. Beerman[a], Vaibhav Jain[c], Henry K. E. Ohman[a,b], Brandon A. Thompson[a,b], Karen R. Abramson[c], Dennis C. Ko[a], Simon G. Gregory[c], Clare M. Smith[a], Jadee L. Neff[d], Rebecca J. Richardson[e], Jason E. Stout[f], David M. Tobin[a,b,#].

a. Department of Molecular Genetics and Microbiology, Duke University School of Medicine, Durham, NC 27710, USA.
b. Department of Integrative Immunobiology, Duke University School of Medicine, Durham, NC, USA.
c. Duke Molecular Physiology Institute, Duke University, Durham, NC 27710, USA.
d. Department of Pathology, Duke University School of Medicine, Durham, NC 27710, USA.
e. School of Physiology, Pharmacology and Neuroscience, University of Bristol, Biomedical Sciences Building, University Walk, Bristol BS8 1TD, UK.
f. Department of Medicine, Division of Infectious Diseases, Duke University School of Medicine, Durham, NC 27710, USA
# Address correspondence to Charlie J. Pyle, charlie.pyle@duke.edu, or David M. Tobin, david.tobin@duke.edu

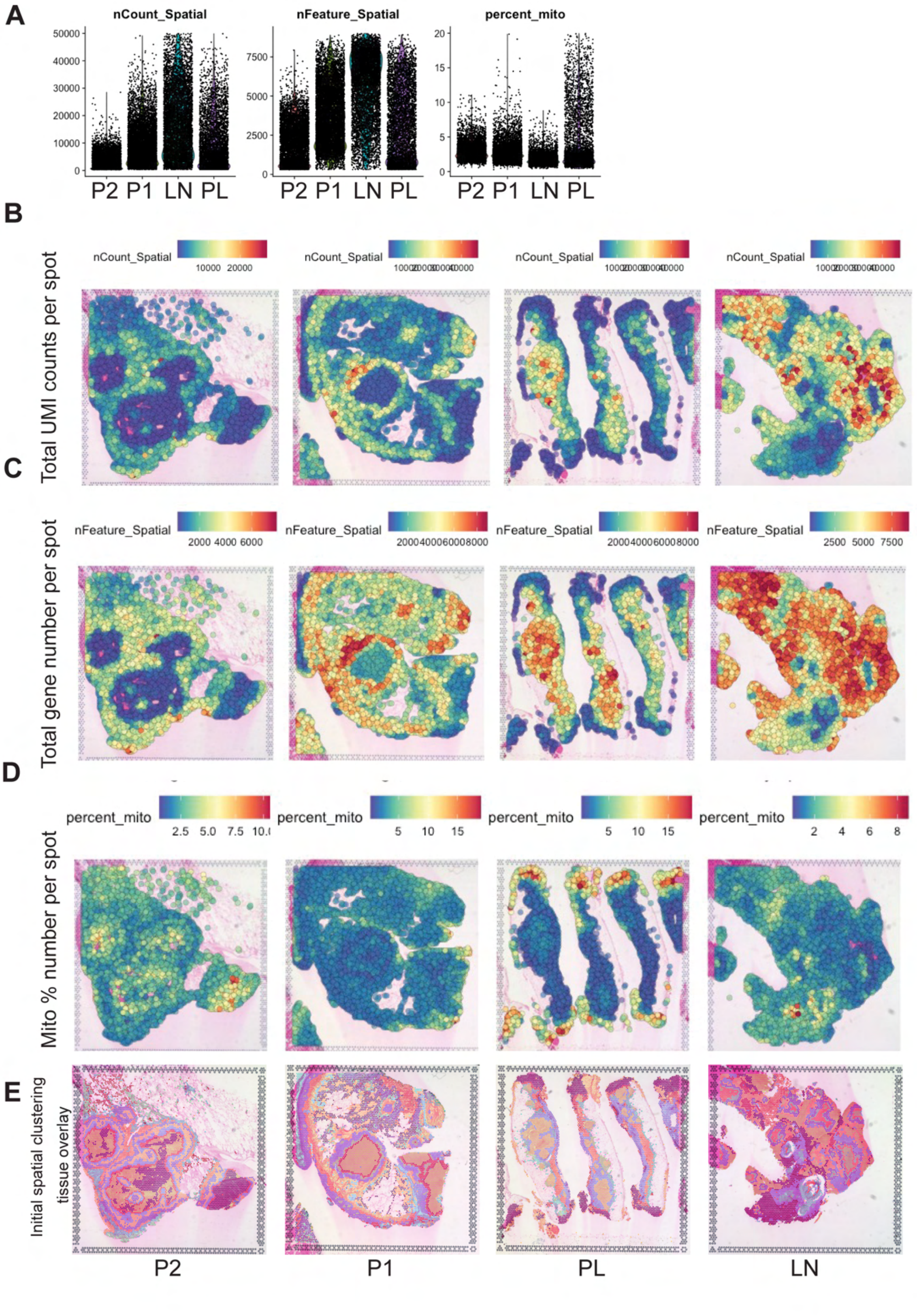

Figure S1: Quality control of Visium v2 spatial assays represented as (A) Violin dot plots for total mRNA reads per cell (left), the number of gene detected per cell (middle), and percentage of reads mapped to mitochondrial gene per cell (right) or (B) (C) (D) heatmaps of spatial distribution. (E) The overlay of initial spatial clustering on Eosin-stained tissue sections.

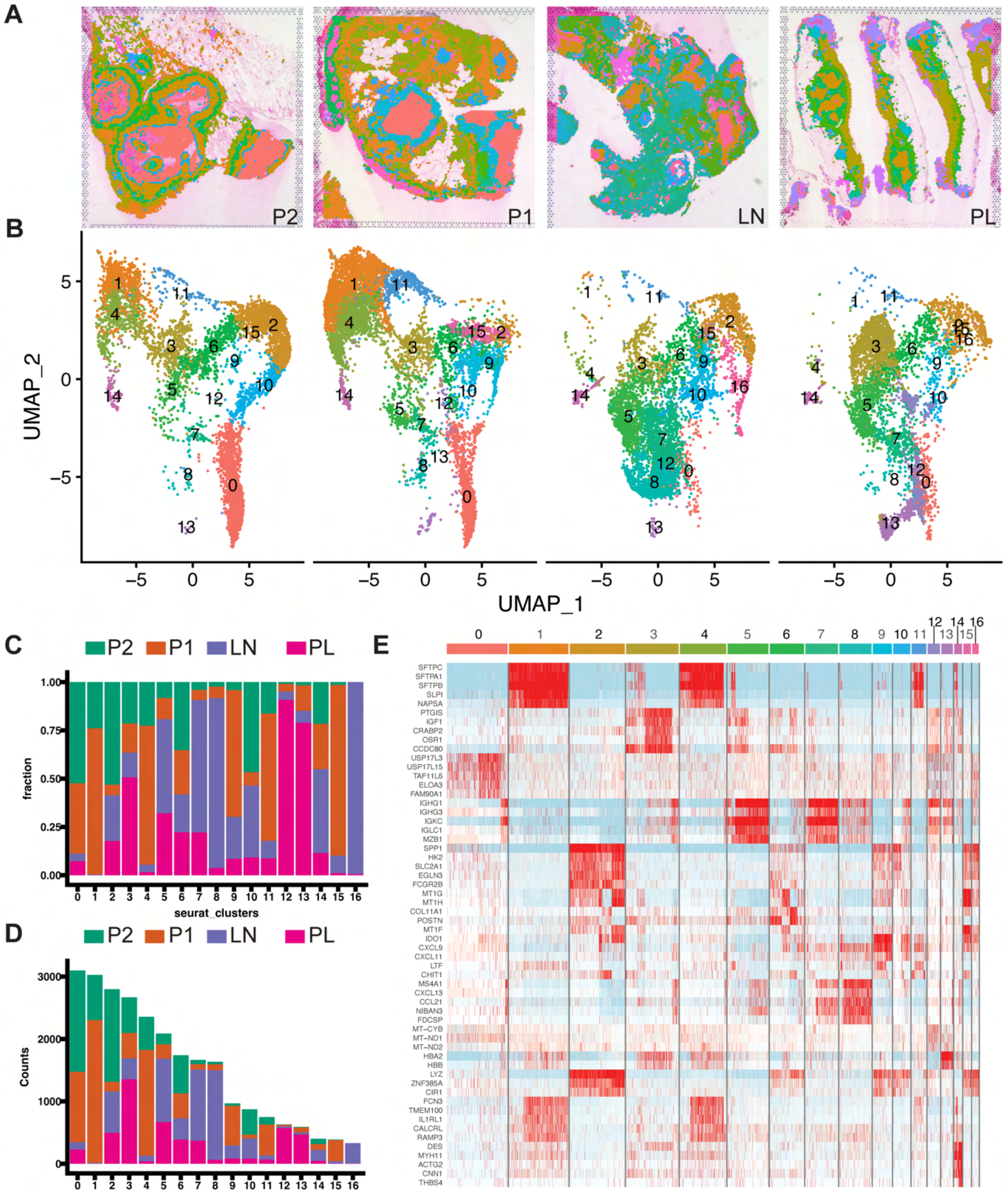

Figure S2: Variability of conserved clusters between granulomas. (A) Visium v2 spot and (B) UMAP plots show the unsupervised cell clusters for each sample. Bar charts representing (C) the relative fraction of individual samples for each cluster and (D) the number of cells from each sample for each cluster. (E) A heatmap plot of the top 5 differentially expressed genes for each spatial cluster.

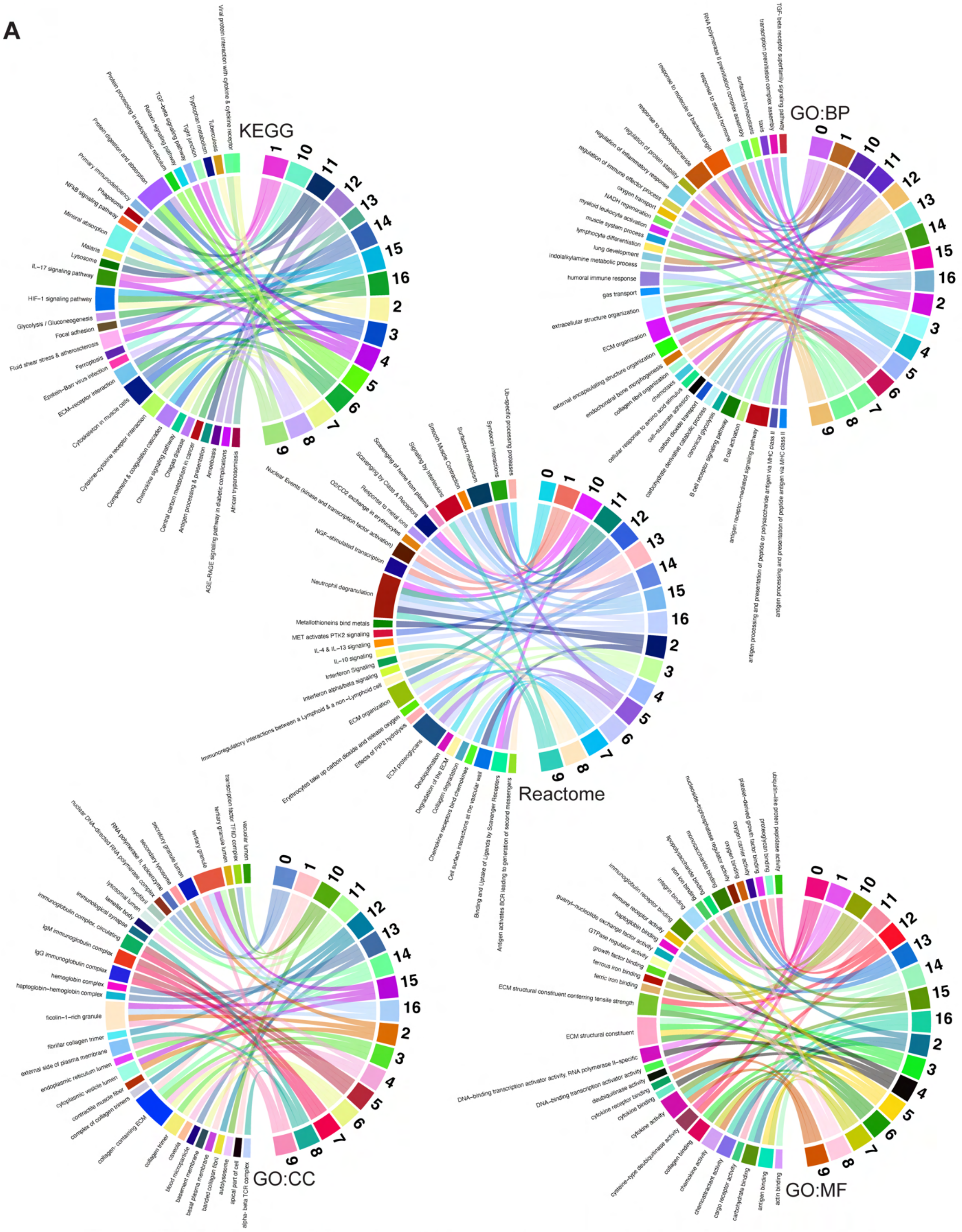

Figure S3: Spatial activity inference in granulomas (A) Chord plots of the top 3 pathways enriched in spatial clusters by over-representation analysis of differentially expressed genes (adj. p < 5x10-5 and log2FC > 0.5) using KEGG, Reactome and Gene Ontogeny (Biological Process, Molecular Function and Cellular component) databases filtered using an adj. p < 5x10-7.

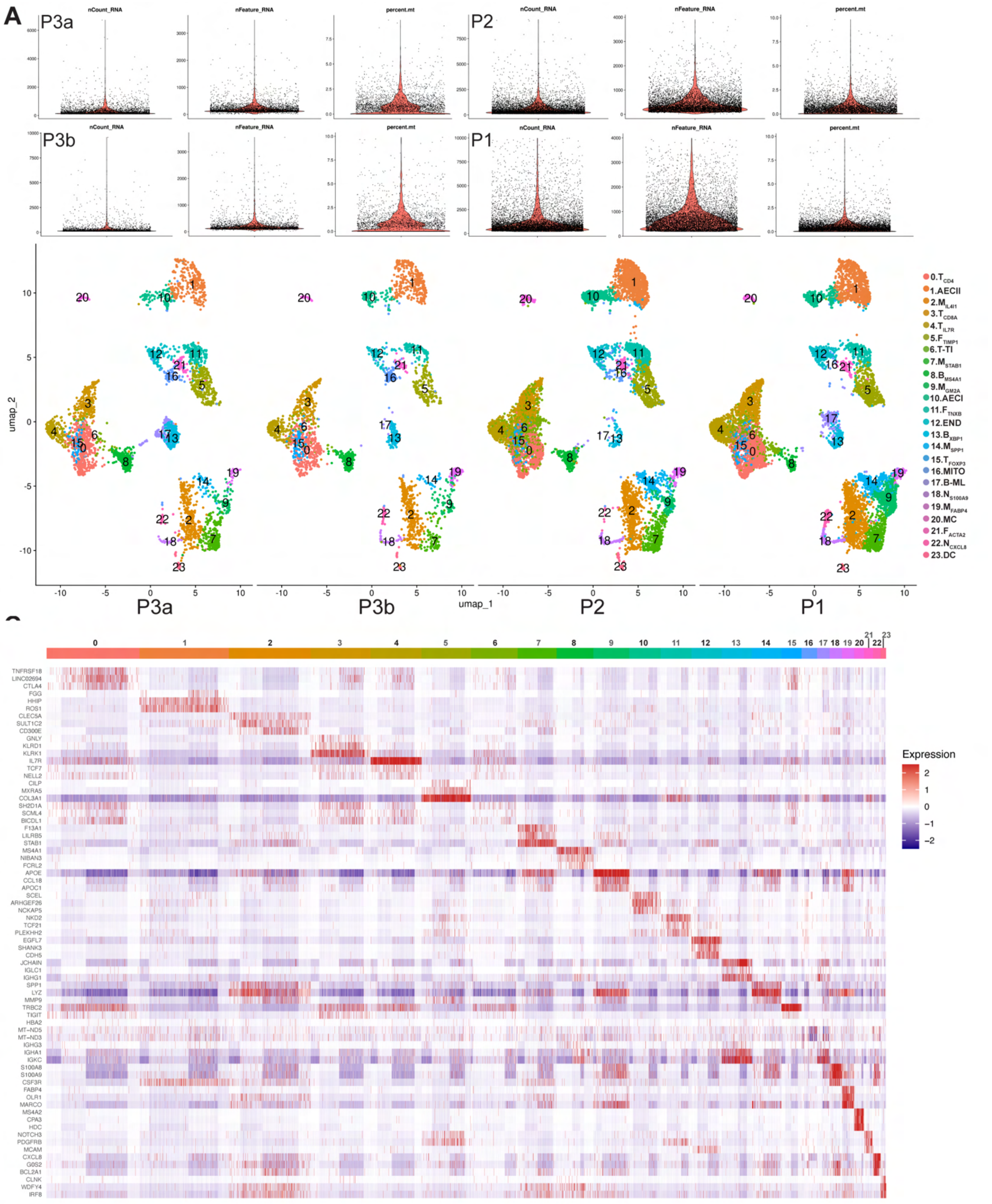

Figure S4: Distribution of tissue parameters in individual samples used in granuloma single-cell Seq assays represented as (A) Violin dot plots for quality control analysis or (B) UMAP plots of cluster distributions (B). (C) A heatmap plot of the normalized expression values for the top 3 differentially expressed genes for each cluster (cell-type) in the normalized single-cell dataset.

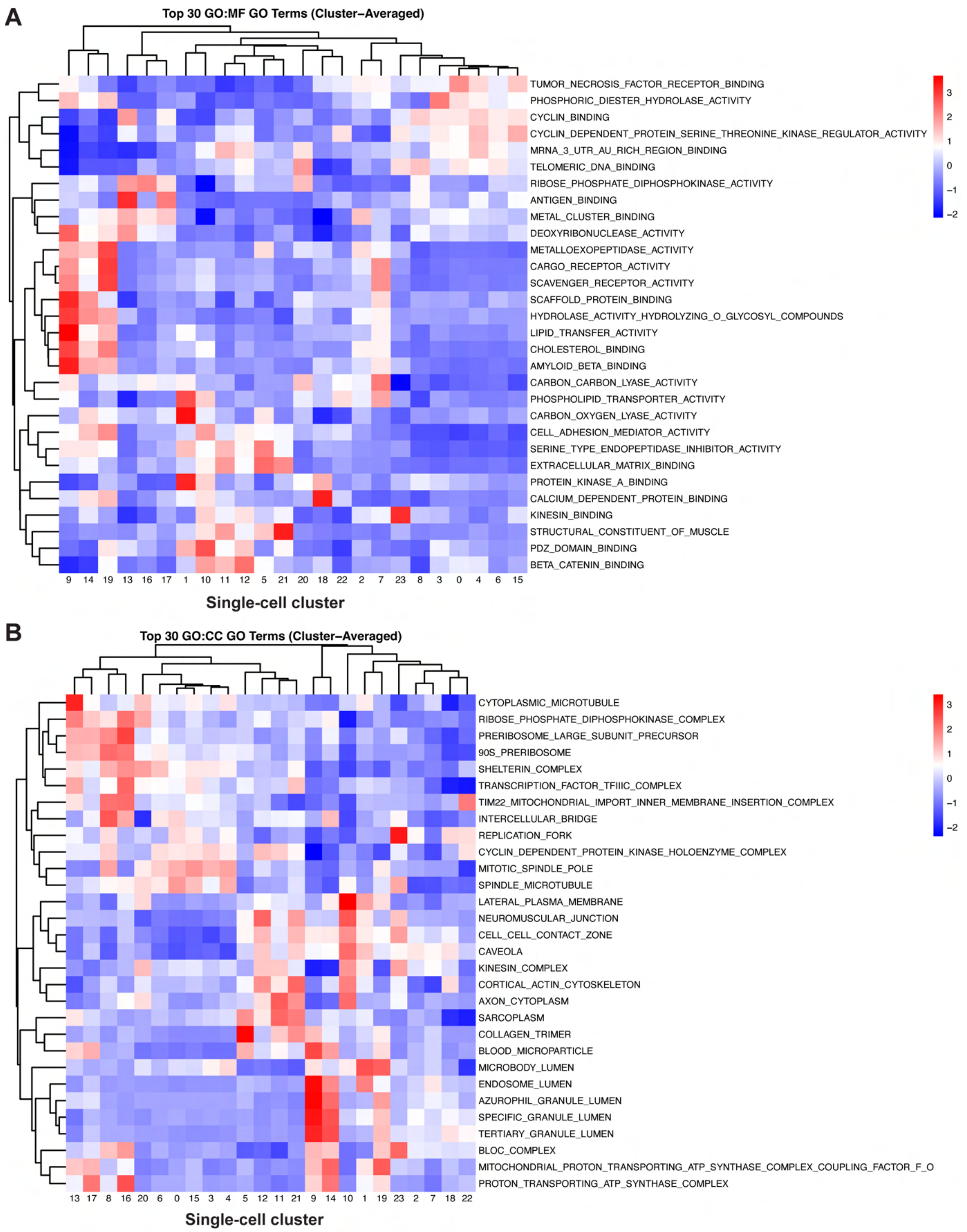
A
Top 30 GO:MF GO Terms (Cluster–Averaged)
TUMOR_NECROSIS_FACTOR_RECEPTOR_BINDING
PHOSPHORIC_DIESTER_HYDROLASE_ACTIVITY
CYCLIN_BINDING
CYCLIN_DEPENDENT_PROTEIN_SERINE_THREONINE_KINASE_REGULATOR_ACTIVITY
MRNA_3_UTR_AU_RICH_REGION_BINDING
TELOMERIC_DNA_BINDING
RIBOSE_PHOSPHATE_DIPHOSPHOKINASE_ACTIVITY
ANTIGEN_BINDING
METAL_CLUSTER_BINDING
DEOXYRIBONUCLEASE_ACTIVITY
METALLOEXOPEPTIDASE_ACTIVITY
CARGO_RECEPTOR_ACTIVITY
SCAVENGER_RECEPTOR_ACTIVITY
SCAFFOLD_PROTEIN_BINDING
HYDROLASE_ACTIVITY_HYDROLYZING_O_GLYCOSYL_COMPOUNDS
LIPID_TRANSFER_ACTIVITY
CHOLESTEROL_BINDING
AMYLOID_BETA_BINDING
CARBON_CARBON_LYASE_ACTIVITY
PHOSPHOLIPID_TRANSPORTER_ACTIVITY
CARBON_OXYGEN_LYASE_ACTIVITY
CELL_ADHESION_MEDIATOR_ACTIVITY
SERINE_TYPE_ENDOPEPTIDASE_INHIBITOR_ACTIVITY
EXTRACELLULAR_MATRIX_BINDING
PROTEIN_KINASE_A_BINDING
CALCIUM_DEPENDENT_PROTEIN_BINDING
KINESIN_BINDING
STRUCTURAL_CONSTITUENT_OF_MUSCLE
PDZ_DOMAIN_BINDING
BETA_CATENIN_BINDING
9 14 19 13 16 17 1 10 11 12 5 21 20 18 22 2 7 23 8 3 0 4 6 15
Single-cell cluster
3 2 1 0 −1 −2
B
Top 30 GO:CC GO Terms (Cluster–Averaged)
CYTOPLASMIC_MICROTUBULE
RIBOSE_PHOSPHATE_DIPHOSPHOKINASE_COMPLEX
PRERIBOSOME_LARGE_SUBUNIT_PRECURSOR
90S_PRERIBOSOME
SHELTERIN_COMPLEX
TRANSCRIPTION_FACTOR_TFIIIC_COMPLEX
TIM22_MITOCHONDRIAL_IMPORT_INNER_MEMBRANE_INSERTION_COMPLEX
INTERCELLULAR_BRIDGE
REPLICATION_FORK
CYCLIN_DEPENDENT_PROTEIN_KINASE_HOLOENZYME_COMPLEX
MITOTIC_SPINDLE_POLE
SPINDLE_MICROTUBULE
LATERAL_PLASMA_MEMBRANE
NEUROMUSCULAR_JUNCTION
CELL_CELL_CONTACT_ZONE
CAVEOLA
KINESIN_COMPLEX
CORTICAL_ACTIN_CYTOSKELETON
AXON_CYTOPLASM
SARCOPLASM
COLLAGEN_TRIMER
BLOOD_MICROPARTICLE
MICROBODY_LUMEN
ENDOSOME_LUMEN
AZUROPHIL_GRANULE_LUMEN
SPECIFIC_GRANULE_LUMEN
TERTIARY_GRANULE_LUMEN
BLOC_COMPLEX
MITOCHONDRIAL_PROTON_TRANSPORTING_ATP_SYNTHASE_COMPLEX_COUPLING_FACTOR_F_O
PROTON_TRANSPORTING_ATP_SYNTHASE_COMPLEX
13 17 8 16 20 6 0 15 3 4 5 12 11 21 9 14 10 1 19 23 2 7 18 22
Single-cell cluster
3 2 1 0 −1 −2

C

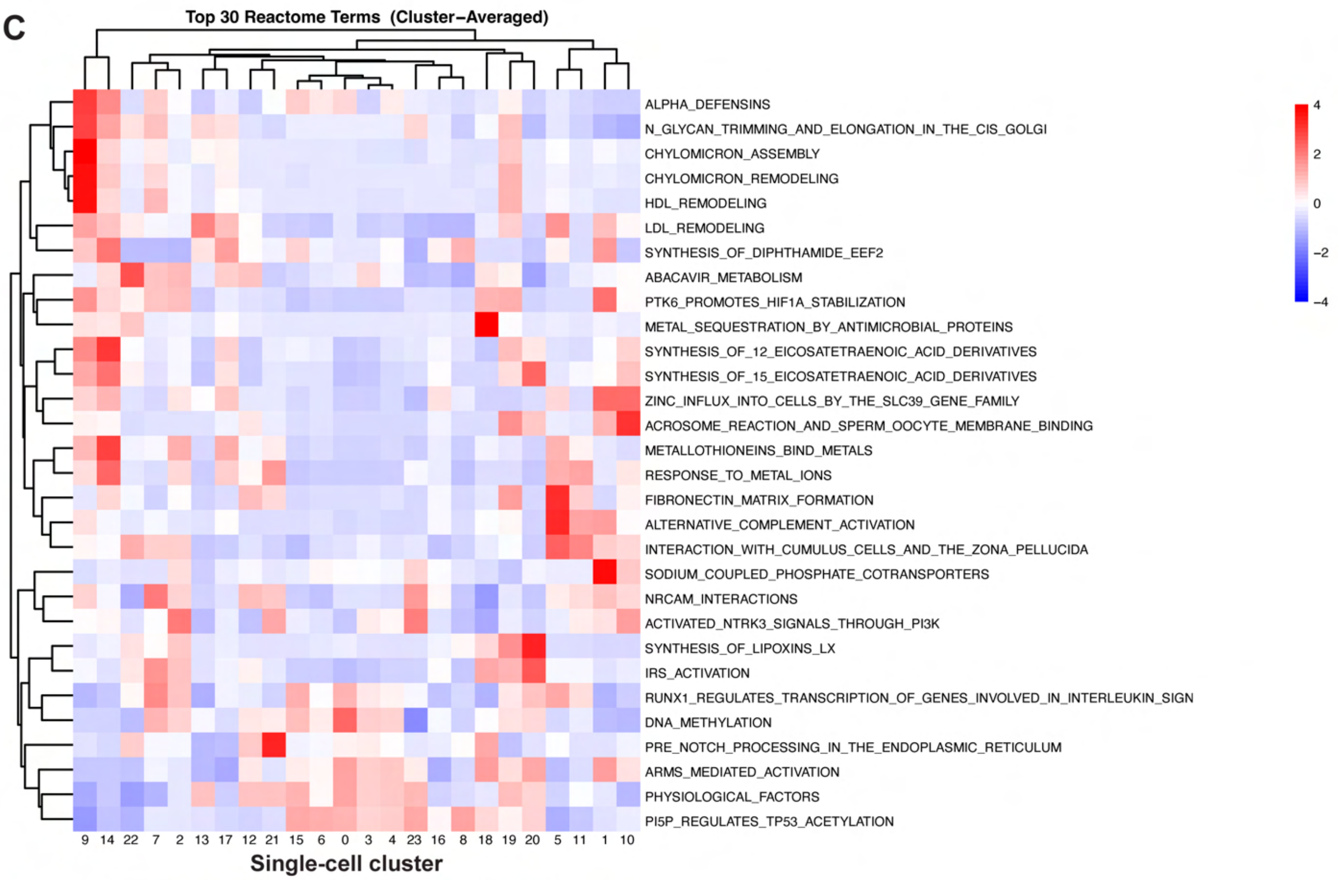

D

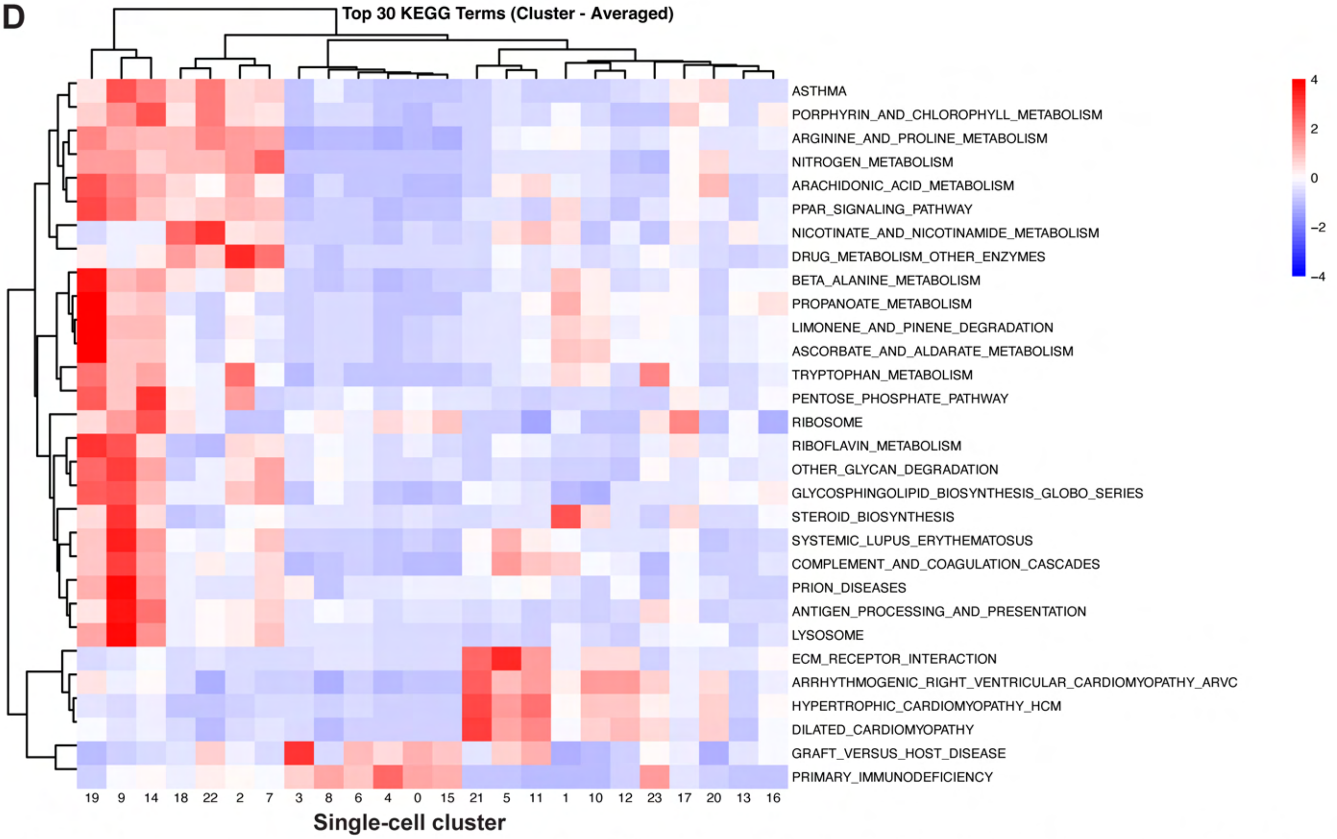

Figure S5: Inference of granuloma cell-type function in the single-cell dataset. Heatmap plots of gene set variation analysis, calculated using median absolute deviation, were used to determine pathway enrichment for the top 30 enriched pathways using the Gene ontology database to determine (A) molecular functions and (B) cellular components or (C) the top 30 enriched pathways using the Reactome database or (D) the top 30 enriched pathways using the KEGG database.
