# Supplementary material for: Paired single-cell and spatial transcriptional profiling reveals a central osteopontin macrophage response mediating tuberculous granuloma formation
Source: mBio. 2025 Aug 7;16(9):e01559-25. doi: 10.1128/mbio.01559-25 (PMC12421895; doi:10.1128/mbio.01559-25)
Supplement: Supplemental Figures, part II — Figures S6 to S8. [file mbio.01559-25-s0002.pdf]

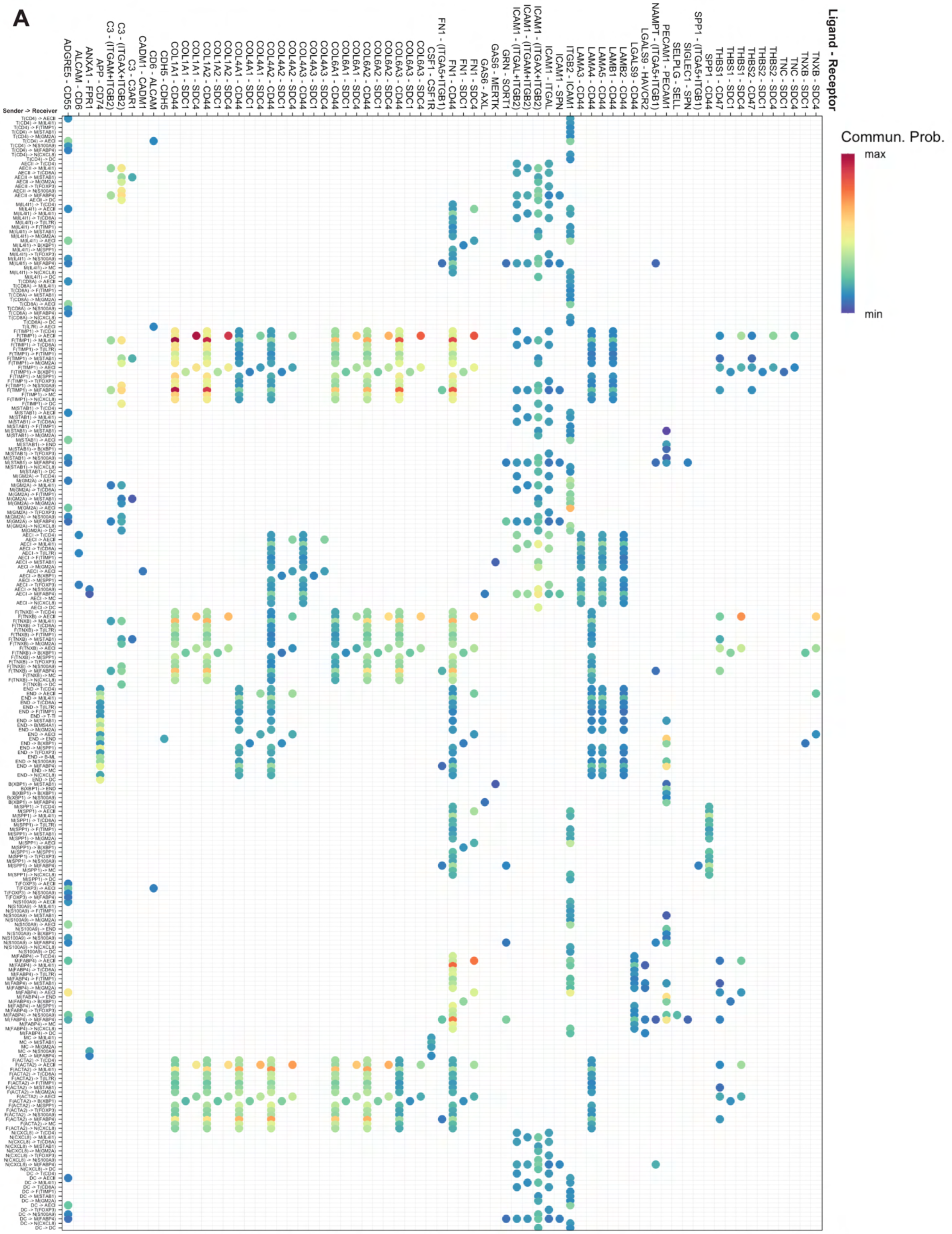

Figure S6: (A) A bubble plot generated using CellChat, showing the communication probability score of receptor-ligand interactions (X-axis) between sender and receiver cell-type combinations (Y-axis).

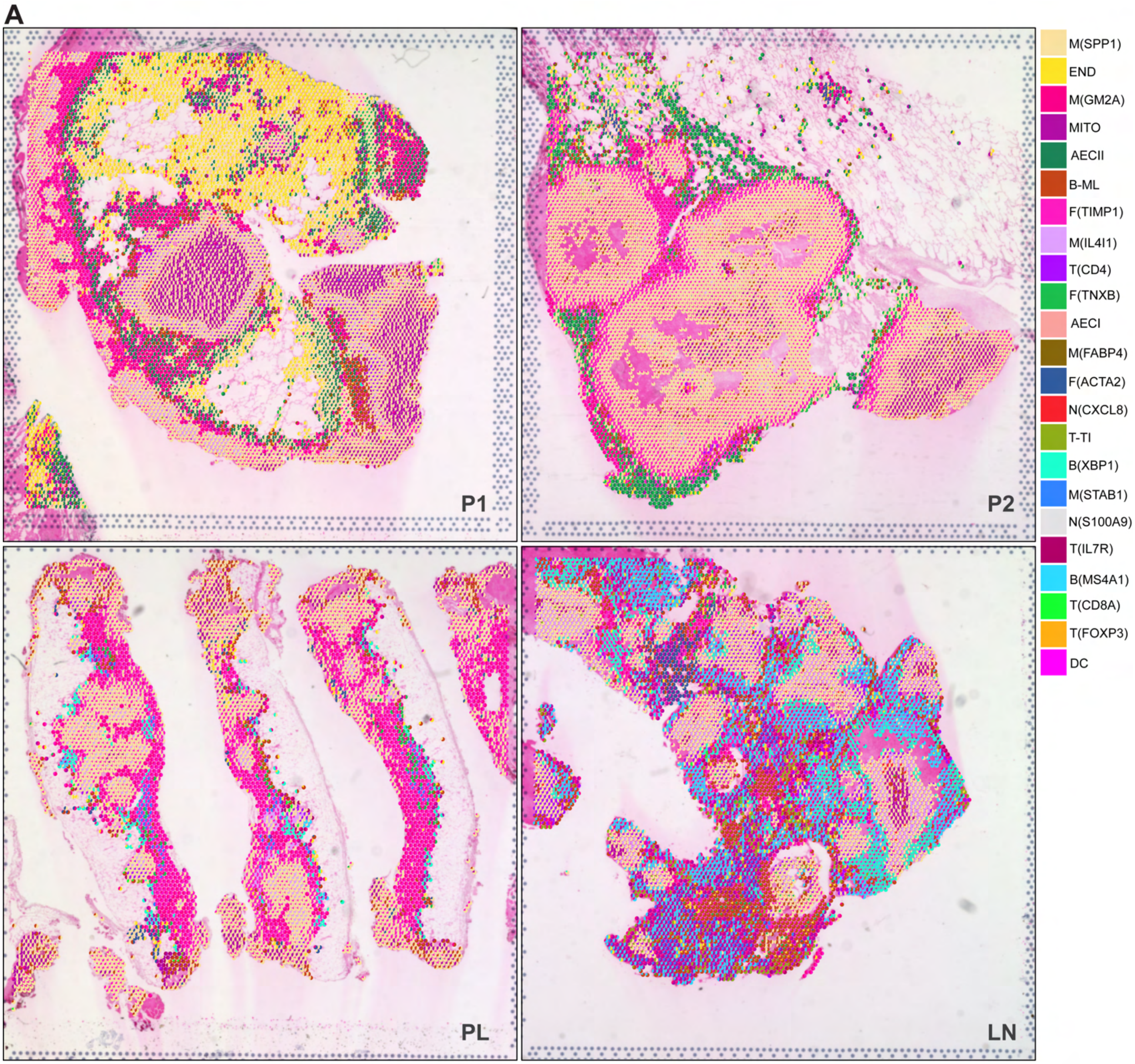

Figure S7: The deconvolution of spatial and single-cell clusters provides a spot-by-spot blueprint of cell-type specific regional occupancy in human *M. tb* granulomas. (A) Pie charts for individual spots from RCTD deconvolution analysis show cell type composition of the primary-major and secondary-minor cell-type occupancy of each 55 µM Visium v2 spot.

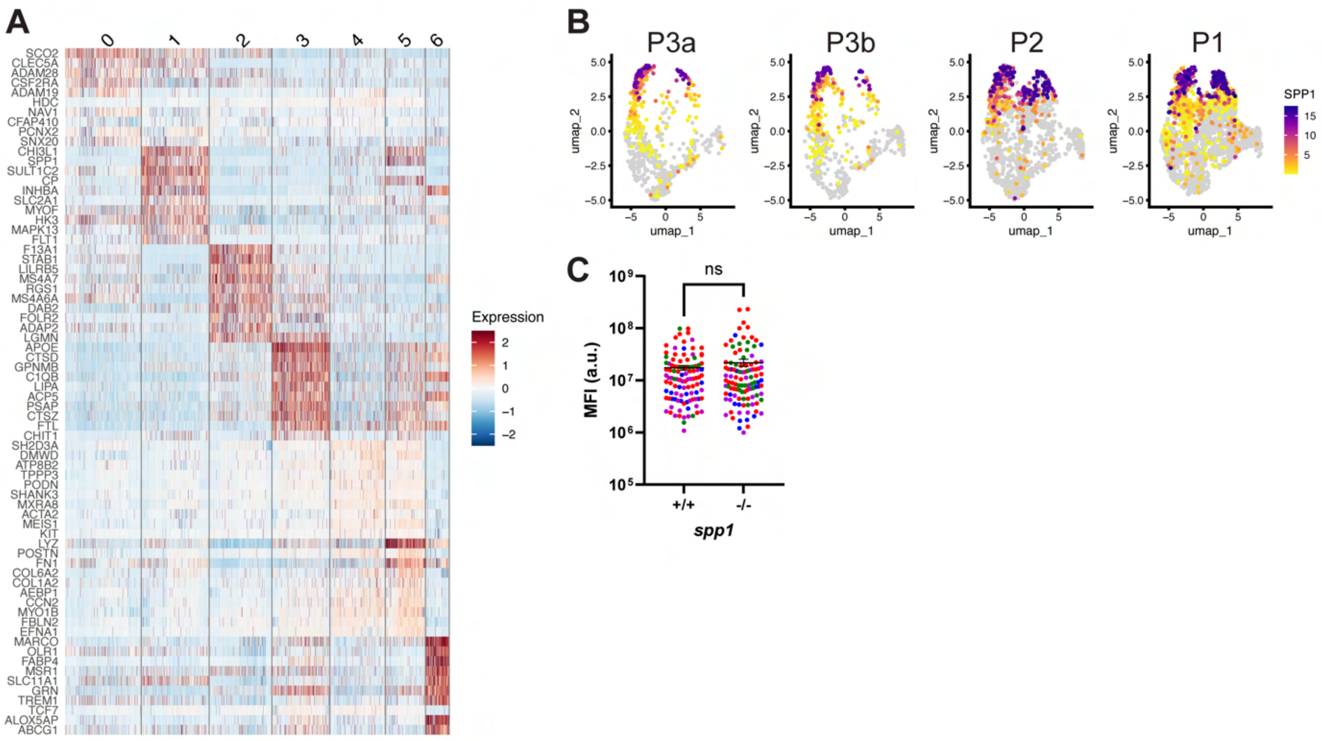

Figure S8: SPP1 in granuloma macrophages. (A) A heatmap plot showing the relative expression levels of top differentially expressed genes across macrophage clusters. (B) A UMAP heatmap plot showing *SPP1* expression in macrophage populations from individual single-cell samples. (C) *M. marinum* larval burden as determined by bacterial fluorescence is not different between control and spp1-knockout animals at 4 days post infection

Table S1: The differentially expressed genes unique to each of the 17 regions in TB granuloma tissue biopsy specimens P1, P2, PL and LN that have a common transcriptional signature between samples as determined by 10x Genomics Visium v2 spatial mRNA sequencing.

Table S2: The normalized average expression values and differentially expressed genes for each of the 24 clusters identified in TB granuloma tissue biopsy specimens P1, P2, P3a and P3b as determined by Illumina single-cell mRNA sequencing.

Table S3: The differentially expressed genes unique to each of the 7 macrophage populations in TB granuloma tissue biopsy specimens P1, P2, P3a and P3b that have a common transcriptional signature between samples as determined by Illumina single-cell mRNA sequencing and Slingshot lineage inference.

Table S4: The normalized average expression values for infection conditions between macrophages that were infected (IMac) or uninfected (UMac) and were obtained either from larvae infected with green-fluorescent *M. marinum* (IL) or control uninfected larvae (UL) as determined by Illumina bulk mRNA sequencing of flow-sorted red-fluorescent macrophages.
